# Long-read sequencing identifies novel structural variations in colorectal cancer

**DOI:** 10.1371/journal.pgen.1010514

**Published:** 2023-02-22

**Authors:** Luming Xu, Xingyue Wang, Xiaohuan Lu, Fan Liang, Zhibo Liu, Hongyan Zhang, Xiaoqiong Li, ShaoBo Tian, Lin Wang, Zheng Wang

**Affiliations:** 1 Department of Clinical Laboratory, Union Hospital, Tongji Medical College, Huazhong University of Science and Technology, Wuhan, China; 2 Research Center for Tissue Engineering and Regenerative Medicine, Union Hospital, Tongji Medical College, Huazhong University of Science and Technology, Wuhan, China; 3 Department of Gastrointestinal Surgery, Union Hospital, Tongji Medical College, Huazhong University of Science and Technology, Wuhan, China; 4 GrandOmics Biosciences, Beijing, China; Radboudumc, NETHERLANDS

## Abstract

Structural variations (SVs) are a key type of cancer genomic alterations, contributing to oncogenesis and progression of many cancers, including colorectal cancer (CRC). However, SVs in CRC remain difficult to be reliably detected due to limited SV-detection capacity of the commonly used short-read sequencing. This study investigated the somatic SVs in 21 pairs of CRC samples by Nanopore whole-genome long-read sequencing. 5200 novel somatic SVs from 21 CRC patients (494 SVs / patient) were identified. A 4.9-Mbp long inversion that silences APC expression (confirmed by RNA-seq) and an 11.2-kbp inversion that structurally alters CFTR were identified. Two novel gene fusions that might functionally impact the oncogene RNF38 and the tumor-suppressor SMAD3 were detected. RNF38 fusion possesses metastasis-promoting ability confirmed by *in vitro* migration and invasion assay, and *in vivo* metastasis experiments. This work highlighted the various applications of long-read sequencing in cancer genome analysis, and shed new light on how somatic SVs structurally alter critical genes in CRC. The investigation on somatic SVs via nanopore sequencing revealed the potential of this genomic approach in facilitating precise diagnosis and personalized treatment of CRC.

## Author summary

Structural variants contribute to oncogenesis and progression of colorectal cancer, but they remain difficult to be reliably detected. Aiming at obtaining a comprehensive picture of somatic SVs in CRC, we perform long-read nanopore sequencing on CRC tumor samples and their matched para-carcinoma tissues. Our results show long-read sequencing precisely and reliably detects 494 somatic SVs per sample, which are significantly more than previously reported short-read sequencing based studies. We find large scale inversions (>10 kbp) that are frequently difficult to be detected by short-read sequencing and alter the expression or structure of key tumor suppressor genes (including APC and CFTR). A novel gene fusion RNF38-RAD51B is also identified, and we find it functionally acts to enhance migration, invasion, and metastasis capabilities of colorectal cancer cells. Although the molecular mechanisms and clinical relevance of the inversions and gene fusions need to be further studied, our work presents a relatively complete SV landscape of CRC, and providing a genetic basis for CRC’s personalized medicine.

## Introduction

Colorectal cancer (CRC) is the third most common malignancy with over 1.8 million new cases and 0.86 million deaths worldwide in 2018 [1]. The development and progression of CRC are largely attributed to genetic alterations, such as structural variations (SVs), single nucleotide variations (SNVs), and epigenetic changes. Among these genetic alterations, the SVs that affect gene expression and function via gene amplification or deletion, gene structure disruption, and gene fusion, are prevalent in CRC [2,3], and have been examined in several studies by copy number variation (CNV) arrays and short-read sequencing [4–7]. These studies identified copy number alterations of oncogenes (including *KRAS* and *MYC*), deletions of tumor suppressors (such as *FHIT*, *PTEN*, *SMAD2* and *SMAD4*), and recurrent R-spondin fusions [8,9]. However, CNV arrays are incapable of determining precise positions of most of SVs, and short-reads sequencing is inefficient in detecting long, complex, or repetitive-region located SVs [10–12]. Thus, precise and detailed detection of SVs in CRC still remains as a challenge [13,14].

Long-read sequencing technologies can generate long continuous reads (length over tens of kilobase pairs (kbp)), possess increased reliability and sensitivity in SVs detection [15]. Pacific Bioscience (as called single-molecule read-time (SMRT) sequencing or PacBio sequencing) and Oxford Nanopore Technologies (ONT, or nanopore sequencing) are the two major strategies of long-read sequencing [16]. Unlike short-read sequencing, PacBio and nanopore sequencing generate reads directly from native DNA (without ultrasonic / enzymatic fragmentation and PCR amplification), avoid the difficulty of detecting variants in genome regions with repeat content or atypical GC content [17]. The advantages of long-read sequencing in studying human diseases were highlighted in several studies. For instance, a pentanucleotide repeat expansion in *SAMD12* that may cause familial cortical myoclonic tremor with epilepsy was identified using nanopore sequencing [18]. This type of repetitive-region residing SVs were difficult to be analyzed by short-read sequencers [18]. In addition to this, long read sequencing identified the leukoencephalopathy-related GGC repeat expansions, X-linked Dystonia-Parkinsonism-related SINE-VNTR-Alu retrotransposon insertions [19], and these variants were previously missed by short read sequencing. Moreover, long read sequencing realized fast and low-cost genome sequencing of pathogen, such as SARS-CoV-2 [20].

In addition to hereditary disease, long-read sequencing also facilitates the studies of cancer genome. Using nanopore sequencing, a complex *KLHDC2-SNTB1* fusion (larger than 10 kbp) composed by three separate chromosome regions was discovered in a breast cancer cell line (SK-BR-3) using nanopore sequencing [11]. In lung adenocarcinoma, a novel class of complex SVs consisting of several small/ middle-sized SVs, were identified via the latest nanopore PromethION sequencer [21]. Given the advantages of long read sequencing, novel large-scale and / or complex SVs that affecting the structure and expression of key oncogenes or tumor suppressor genes, repetitive-region residing SVs that may causing genomic instability (such as transposable element) or contributing to tumor progression, and tumor-promoting gene fusions may be efficiently detected, which would provide a more comprehensive understanding of the genomic aberrations of CRC and further in-depth study of their biology functions.

Here, using long-read whole genome sequencing to analyze CRC tumors from 21 patients, we (1) precisely and reliably detected somatic SVs across the cancer genomes, (2) showed the representative large-scale inversions that altered the expression or structure of key tumor suppressor genes, such as *APC* and *CFTR*, in CRC; (3) discovered a novel gene fusion *RNF38-RAD51B* that could increase the migration, invasion and metastasis ability of CRC cells.

## Material and methods

### Ethics statement

This study was conducted according to the Helsinki human subject doctrine and was approved by the Huazhong University of Science and Technology review board and Ethics Committee (IORG No. IORG0003571, 2020-S197), written consents to participate was acquired from all the patients.

### Sample collection and Oxford nanopore sequencing

21 pairs of tumor samples and matched para-carcinoma samples were obtained from the surgically removed tumor tissues and adjacent intestinal tissues (>6 cm from tumor tissues) of CRC patients in Wuhan Union Hospital, and stored at -80°C. All the samples were analyzed and sequenced using long-read Nanopore sequencing, short-read whole exome sequencing and RNA sequencing. Genomic DNA from each sample was extracted by sodium dodecyl sulphate method. DNA was shared to > 20kb by Covaris g-tude. Then genomic DNA libraries were constructed according to the manufacturer’s instructions by using the Ligation Sequencing kit 1D (SQK-LSK109). The prepared libraries were loaded into R9.4(1D) flow cells and then sequenced on the PromethION sequencer (ONT, UK). Then Guppy (version: 2.0.8) was used to perform basecalling on fast5 files to generate FASTQ format files.

### Alignment and SVs calling

All the reads from ONT sequencing were aligned to the human reference genome with only major chromosomes 1–22 and X, Y from NCBI (ftp://ftp-trace.ncbi.nih.gov/1000 genomes/ftp/technical/reference/human_g1k_v37.fasta.gz) using NGMLR (v0.2.7) with default parameters. Samtools (v1.9) was used to compute alignment ratio and mapping identity by analyzing bam files. Structural Variations were called using Sniffles v1.0.8 with minimum reads supporting number 2 and minimum SV size 50bp. In order to obtain high-quality SVs in tumor and normal samples, only SVs supported by at least 0.3 folds of average sequencing depth were retained.

Somatic SVs (present in tumor but not in normal samples SV calls) were obtained by comparing high quality tumor samples SVs passed above filtering thresholds with normal samples SVs only supported by two or more reads. This strategy is to improve the recall rate of normal samples SVs to improve the reliability of somatic SVs. Tumor and matched normal sample SVs were merged using svmerge (https://github.com/GrandOmics/svmerge) with a maximum distance of 1000bp for all types SVs, 40% reciprocal overlaps for deletions, inversions and duplication and difference in SV length less than 20%. We used svhawkeyes (https://github.com/yywan0913/SVhawkeye) for the manual curation of unfiltered somatic SVs. The reads alignment images of each unfiltered somatic SVs were generated by svhawkeyes from alignment files and manually checked. Somatic SVs that appear in both cancer and paired normal samples were identified as false positive. Finally, all the somatic SVs were merged into an integrated call set. SVs with upstream and downstream genes were annotated in the segdup (UCSC golden path hg19), rmsk (UCSC golden path hg19), dgv (2016-05-15), 1000 Genome Project (phase 3), gnomAD (2.1.1), and COSMIC databases (v70) using annovar (2017-07-17). Insertions were further annotated as tandem repeats or known repeat classes using TRF (4.09) and RepeatMasker (4.1).

### Whole exome sequencing and variants calling

Sheared genome DNA from each tumor and normal sample was used for library preparation. Exome DNA was captured using the XGen Exome Research Panel v1.0 51Mb kit and sequenced using the Illumina NovaSeq platform with 150 bp paired-end sequencing mode. The sequencing depth of each sample was above 200X. Bam files were generated using sentieon DNA pipelines (sentieon-genomics-201808.01) including alignment, removing duplications, sorting and local realignment following the Broad Institute’s best practices. Somatic mutations and Indels were detected by using Sentieon TNscope from co-realigned tumor and normal BAM files with dbSNP 138 in target intervals. All somatic mutations and Indels were annotated in the dbSNP 147, clinvar (2017-05-01), ExAC (2016-04-23), 1000 Genome Project (phase 3), gnomAD (2.1.1), InterVar (2017-02-02) and COSMIC databases (v70) using ANNOVAR (2017-07-17).

### Transcriptome sequencing and quantification of gene expression level

Sequencing libraries were generated using NEBNext Ultra RNA Library Prep Kit for Illumina (NEB, USA) according to the manufacturer’s instructions. AMPure XP system (Beckman Coulter, Beverly, USA) was used to purify the library fragments and 3 μl USER Enzyme (NEB, USA) was used for size selection (250~300 bp). The library preparations were sequenced on an Illumina Hiseq platform with 150 bp paired-end model, and at least 6 G of clean data were generated for each sample. Paired-end reads were aligned to the reference genome using Hisat2 (v2.0.5). Reads counts were calculated using FeatureCounts (v1.5.0-p3). Differential expression analysis was performed using the edgeR R package (v 3.18.1) and significance was defined as adjusted P-value < 0.05 and foldchange > 2.

### Novel gene fusions identification

Fusion gene usually caused by reasons such as chromosome translocation, inversion and deletion. Two genes containing two breakpoints of the same SV respectively were selected as candidate fusion gene. star-fusion(1.2.0) was used to detect fusion genes from Illumina RNA sequencing data with—annotate;—examine_coding_effect;—FusionInspector inspect;—denovo_reconstruct;—min_junction_reads 1;—min_sum_frags 2. Fusion genes predicted by structural variation and expressed in the RNAseq data were further used for manual curation from both nanopore whole genome sequencing and Illumina RNA sequencing alignments. Primers were designed to span fusion junction and were validated by PCR and Sanger sequencing.

#### Primer sequences

**Table:** 

	Breakpoint 1		Breakpoint 2	
	Forward (5’ to 3’)	Reverse (5’ to 3’)	Forward (5’ to 3’)	Reverse (5’ to 3’)
APC inversion	TGGGTATCAGATCTCTATAGGCTGT	GCACTTCTATGTATGTGTCAGGG	ACCAGAAGGCAGGGTCATTG	CCCAGCAAGCAAGGAAGTTG
CFTR inversion	ACAAATTCCAAGACTTACTGGCA	TGGTCACTGGCTTGTTGAGA	GACATGATCCTTTTGCAGCCT	TGTGCCCACAGTTCAAACCT
RNF38-RAD51B gene fusion	TGGTTTGGCTACTTTCCCTCT	GCAGGGGTACTCAAAGTCCC		
SMAD3-SHISA6 gene fusion	GAAGCCAAAACACCGGACAC	TCATACTTCTGGGGCTGGGA	TGGCTGAAGGTCTGTTTTGT	ACAGAGAAGCCAAGAAGCCA

### Cell lines

HEK293T (human embryo kidney cell line), LoVo (human colon adenocarcinoma cell line) and HCT116 (human rectum adenocarcinoma cell line) cells were purchased from the American Type Culture Collection (Rockville, MD, USA) and maintained in Dulbecco’s Modified Eagle medium (Hyclone, Logan, UT, USA), supplemented with 10% fetal bovine serum (Sciencell, Carlsbad, CA, USA) at 37°C under 5% CO_2_ in a cell incubator.

### Establishment of RNF38-RAD51B overexpressing cell lines

The cDNA of RNF38 and RAD51b was amplified from a cDNA library of HCT116 cells, then cloned into pLenti-puro lentiviral reporter plasmid to form a RNF38-RAD51b overexpression vector. The overexpression vector was confirmed by PCR and Sanger sequencing (sequences of the primer pair are listed below). Then, the lentivirus vector was obtained by co-transfecting HEK293T cells with pLenti-puro-RNF38-RAD51b, psPAX2 packaging, and pMD2.G enveloped plasmids according to the manufacturer’s instructions. HCT116 and LoVo cells were infected by filtered lentivirus (pLenti-puro-vector or pLenti-puro-RNF38-RAD51b) with polybrene (8 μg/mL) and then selected by puromycin (1 μg/mL) for 1 week. The expression level of RNF38-RAD51B fusion gene was measured using western-blot.

### Transwell migration and invasion assays

The migration and invasion assays of RNF38-RAD51B overexpressing HCT116 and LoVo cells were assessed using 8.0-μm pore size transwell inserts. For migration assay, cells were seeded to inserts and cultured for 15 (LoVo cells) or 30 (HCT116 cells) hours. For migration assay, cells were seeded to Matrigel-coated inserts (invasion assay) and cultured for 36 (LoVo cells) or 48 (HCT116 cells) hours Then, the cells on the underside of the inserts were fixed and stained with crystal violet, and counted by microscope. Each experiment was repeated for thrice.

### Animal studies

Six weeks old male BALB/c nude mice purchased from Beijing HFK Bioscience Co., Ltd were used for the animal studies. 1 × 10^6^ RNF38-RAD51B overexpressing HCT116 cells were injected into the livers of the nude mice via the splenic vein (eight mice per group). After six weeks, the mice were euthanized by excessive anesthesia and the livers were collected. Then, the liver tissues were sectioned, stained with hematoxylin and eosin (H&E), and assessed by quantifying the number of metastatic lesions by a microscope.

## Results

### Nanopore sequencing of CRC samples

**Fig 1:**
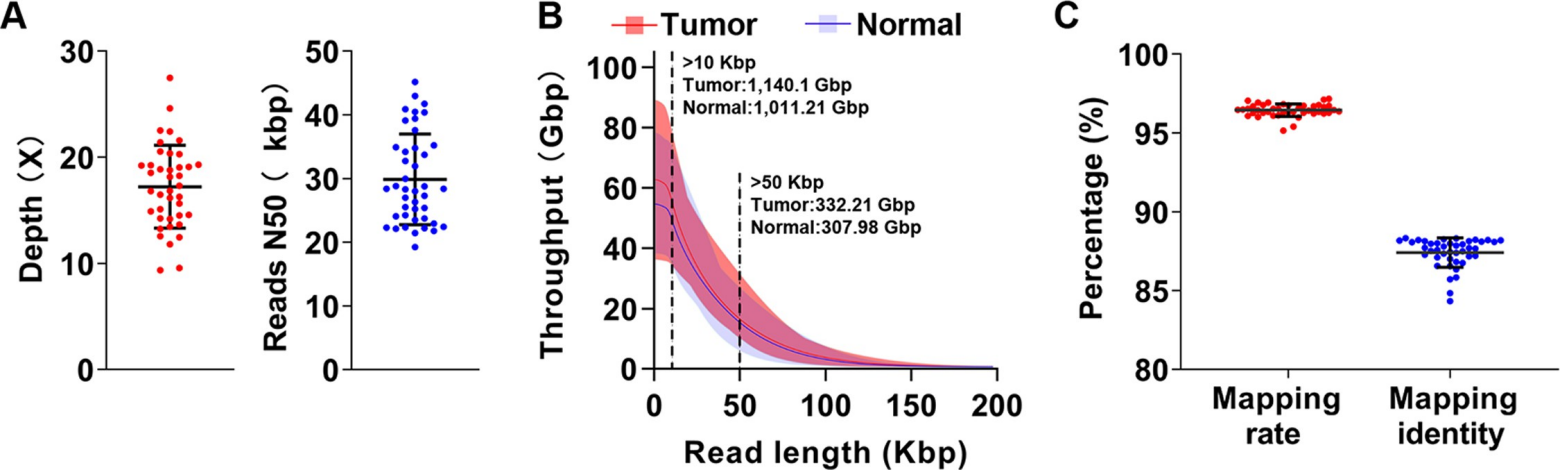
Summary of the long-read sequencing data. **(A)** The sequencing depth (left) and reads N50 (right) of long-read data obtained from 21 pairs of tumor/ normal samples via nanopore sequencer. **(B)** Cumulative distribution of total bases (Y axis) over read length (X axis) for tumor/ normal samples. The quantities of bases in reads 10 Kbp+ and 50 Kbp+ were labeled. **(C)** The mapping rate and identity of long-reads sequencing.

We generated whole-genome long-read sequence data from 21 CRC patients (S1 Table) using PromethION (Oxford Nanopore Technologies) nanopore sequencers. All the patients were at stage II (n = 13) or stage III (n = 8), and five of them were of high-level microsatellite instability (MSI-H). All samples were also analyzed by short-read whole exome sequencing (WES) and RNA-seq to obtain SNVs and gene transcriptional data, respectively. We obtained over 51 billion bases (>17X in depth) long-read data per sample with a mean read N50 of 30,211 bp (range from 19,238 bp to 45,166 bp; 94% of reads were ≥10 kbp) (Fig 1A and 1B and S2 and S3 Tables). The maximum read length and the N50 length of the obtained reads were 897,996 bp and 42,969 bp, respectively, consistent with previously reported PromethION data [21], but longer than those generated by MinION platform [11,15]. With NGMLR [22], 96.3% of the reads were mapped to the reference genome (human G1Kv37) with the mean mapping intensities of 87.4% (Fig 1C).

### Long-read sequencing identified widespread somatic SVs in colorectal cancer

**Fig 2:**
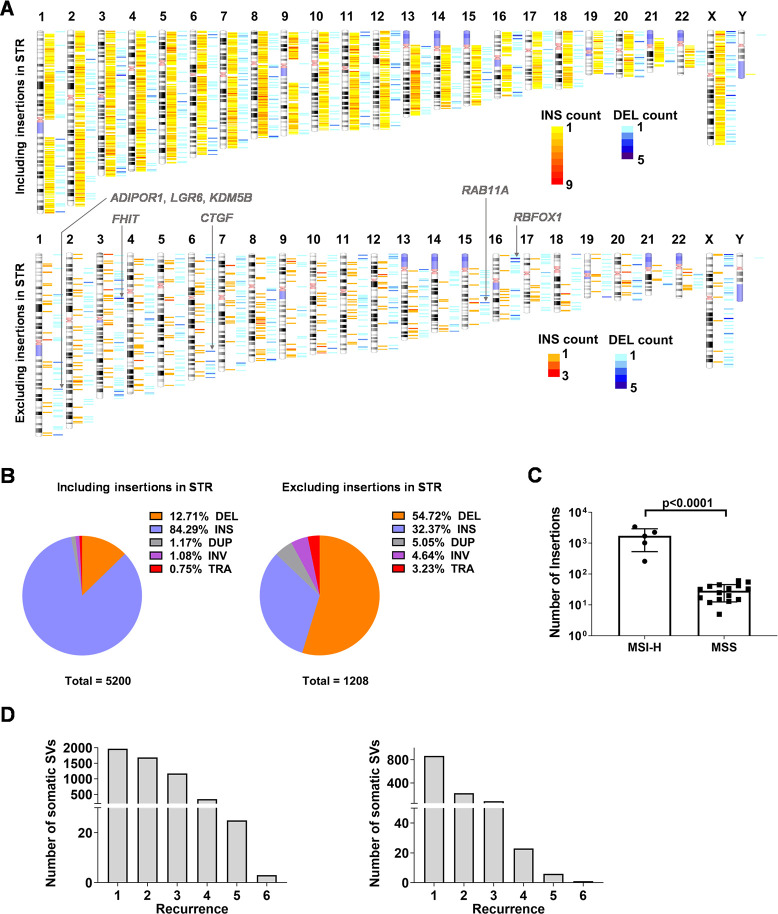
Detection of somatic SVs in CRC by long-read sequencing. **(A)** Chromosome ideogram showing somatic deletions (DEL) and insertions (INS) identified by long-read sequencing in 21 pairs of CRC samples. **(B)** Pie chart showing the percentages of different classes of SVs identified from long-read sequencing data including or excluding insertions in STR regions. **(C)** Quantification of somatic insertions in MSI-H or MSS samples (p<0.0001, Student’s *t*-test). **(D)** The quantities of somatic SVs that were detected in multiple samples, including (left) or excluding insertions in STR regions (right). The “Recurrence” on X-axis refers to the number of samples in which an SV is detected.

We employed Sniffles [22] for SV calling and identified 817,857 SVs in all the samples (19466 SVs per sample) (S1 Fig), largely consistent with previous studies [22,23]. These SVs were used to map somatic SVs, yielding 14508 unfiltered somatic SVs. After manual curation (see Method), we obtained 494 somatic SVs per tumor sample (in total 5,200 nonredundant somatic SVs), significantly more than previous short-read data in CRC [24,25], likely due to the increased sensitivity of long-read sequencing in detecting SVs [11,26]. The lengths of 98% of SVs were less than 10,000 bp, and the mutual distance of most SVs (~80%) was between 10^5^ ~ 10^7^ bp (S2 Fig). The components of these somatic SVs were 661 (12.7%) deletions, 4,383 (84.3%) insertions, 61 (1.2%) duplications, 56 (1.1%) inversions, and 39 (0.8%) translocations (Figs 2A, upper, 2B, left; and S3). The classification of sequences and loci of these insertions and deletions revealed that most of insertions (95%) occur in MSI-H samples due to the abnormal expansion of short tandem repeat (STR) regions (Figs 2C and S4). After exclusion of insertions in STR regions, 54.72%, 32.37%, 5.05%, and 3.32% of somatic SVs were deletions, insertions, duplications, and translocations, respectively (Figs 2A, lower, 2B, right; and S3). The number of inversions in MSI-H samples was significantly lower than that in MSS samples, and the numbers of other types of SVs were similar across different MSI status and different stages (S5 Fig). Some loci with high frequency were associated with the genes involved in oncogenesis and development of CRC, including alternative splicing factor *RBFOX1*, tumor suppressor gene *FHIT*, and several oncogenes such as *LGR6*, *CTGF* and *RAB11A* (Fig 2A). Meanwhile, 62.1% of somatic SVs were detected in at least two samples (Fig 2D). Recurrent insertions were mainly located in STR regions (S6 Fig). In addition, duplications, inversions, and translocations were less likely to be recurrent events, as over 90% of these SVs were singletons (S7 Fig).

### Characterizations of somatic SVs reveal expanded LINE and SINE insertions in CRC

**Fig 3:**
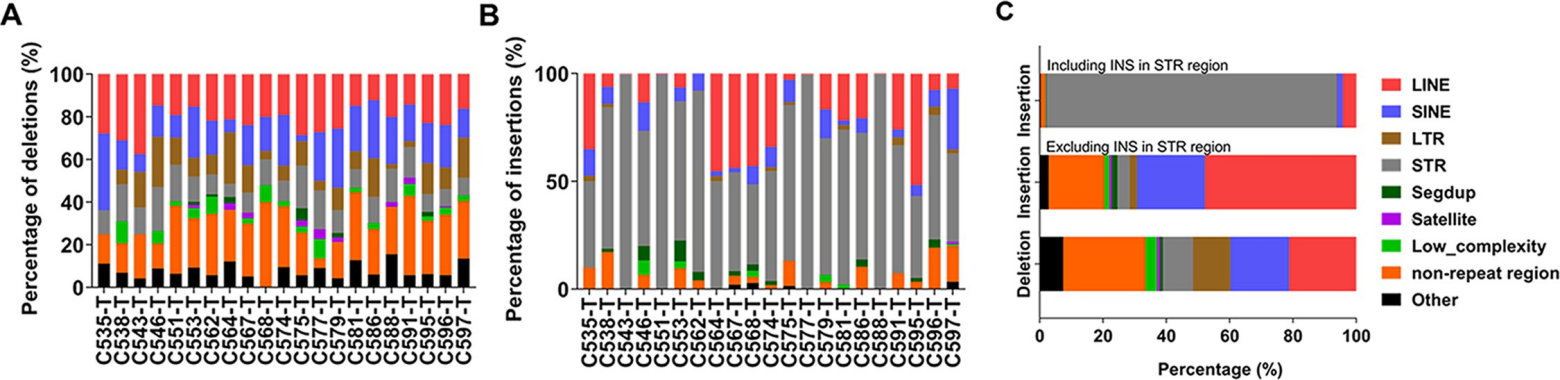
Sequence analysis of somatic deletions/ insertions. **(A** and **B)** The genomic context of somatic deletions (A) and insertions (B) in each sample obtained from sequence analysis using RepeatMasker. Segdup, segment duplications; Satellite, satellite repeats; Low_complexity, low complexity repeats. **(C)** The components and proportions of deletions and insertions including or excluding insertions in STR regions.

We classified the identified SVs by repeat contents of the variant sequence using RepeatMasker (http://www.repeatmasker.org) to explore the genomic context of somatic SVs. Approximately, half of the deletions were located in tandem-repeats regions or mobile elements (for instance, LINE, SINE and Long terminal repeat (LTR)) (Fig 3A). After exclusion of STR regions, approximately 70% of insertions were mobile elements’ insertions (Figs 3B and 3C and S8), half of which were LINE insertions, indicating aberrant activation of LINE-1 retrotransposons in CRC, consistent with previous reports [27,28].

### Large-scale inversions cause dysfunction of tumor suppressors

**Fig 4:**
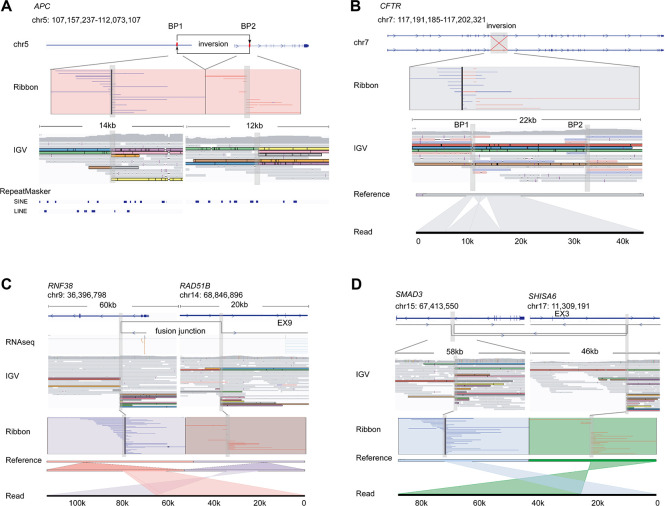
Large-scale inversions and gene fusions detected by nanopore sequencing. **(A-D)** Reads and structures of the 4,915 kbp inversion spanning exon 1 of *APC* (A), the 11.2 kbp inversion in *CFTR* (B), the RNF38-RAD51B gene fusion (C) and the SMAD3-SHISA6 gene fusion (D). For reads alignment, the reads spanning breakpoints were highlighted in colors. For each inversion, the top panel indicates the loci which inversion occurred, and read alignments coving the breakpoints (visualized by Ribbon). The forward and reverse split reads were marked by blue and red, respectively. The middle panel shows reads alignments around breakpoints (visualized by Integrative Genomics Viewer). The bottom panel shows the detailed structure of the SV (visualized by Ribbon). For each gene fusion, the top, middle and bottom panels show translocation loci, reads alignment and split reads, respectively.

In addition to small SVs, large-scale (> 10 kbp) somatic SVs that affected tumor suppressors through disrupting gene structure to silence them, were also detected by nanopore sequencing. In the sample C546-T, a high-confidence 4.9 Mbp inversion that spanned from chr5: 107,157,237 to chr5: 112,073,107 covering the exon 1 of *APC* was identified (Fig 4A). We analyzed the PCR products amplified against the sequences spanning across each breakpoint using Sanger sequencing to detail the structure of both breakpoints (S9A Fig). An 8-bp deletion at breakpoint 1 (BP1) was revealed, which resulted in microhomology and might consequently cause the formation of inversion via microhomology-mediated end joining (S10A Fig). RNA-seq results showed that *APC* expression was sharply decreased (FPKM: 0.296) at mRNA level compared to the paired normal sample (C546-N, FPKM: 2.262). No variant in *APC* was reported using short-read based WES, which was possibly because the base sequence of inverted exon 1 of *APC* was unchanged.

Additionally, we identified an 11.2-kbp somatic inversion that spanned from chr7: 117,191,185 to chr7: 117,202,321 involving the exon 11 of *CFTR* in the sample C564-T (Fig 4B). Notably, four long reads spanned both breakpoints of the inversion (Fig 4B), and covered the complete structure of such a relatively-long inversion. Sanger sequencing of both breakpoints revealed small insertions, deletions, and duplications in the vicinity of both breakpoints, suggesting that this inversion may be generated by microhomology-mediated break-induced replication (S9B and S10B Figs).

### Novel gene fusions identified by long-read sequencing

Long read sequencing have proven immensely helpful in detecting gene fusions [29]. For instance, we identified two new rearrangements that possibly resulted in gene fusions, *RNF38-RAD51B* and *SMAD3-SHISA6*. For *RNF38-RAD51B*, the upstream of the intron 3 of *RNF38* was connected to the downstream of the intron 8 of *RAD51B* (Fig 4C). This gene fusion was also detected by RNAseq and confirmed by PCR products encompassing breakpoint junctions (S9C and S11A Figs). The formation of this fusion might change the function of *RNF38*, which reportedly promotes cancer cell migration and invasion, inhibition of cancer cell apoptosis, and epithelial-mesenchymal transition [30–32]. For *SMAD3-SHISA6* (Fig 4D), PCR validated that the downstream of the intron 7 of *SMAD3* was connected to the upstream of the intron 3 of *SHISA6*, while the upstream of the intron 7 of *SMAD3* was reversely connected to the downstream of intron 7 of *SHISA6* (S9D and S11B Figs). However, this gene fusion was not detected by RNAseq, possibly because of its low expression. Given that SMAD3, a major transcription factor in TGF-β pathway, acts as a tumor suppressor and its functional disruption was positively associated with CRC progression and metastasis [33], this fusion might lead to SMAD3 dysfunction, consequently suppressing the function of TGF-β pathway.

### RNF38-RAD51B promotes CRC cell migration, invasion, and metastasis.

**Fig 5:**
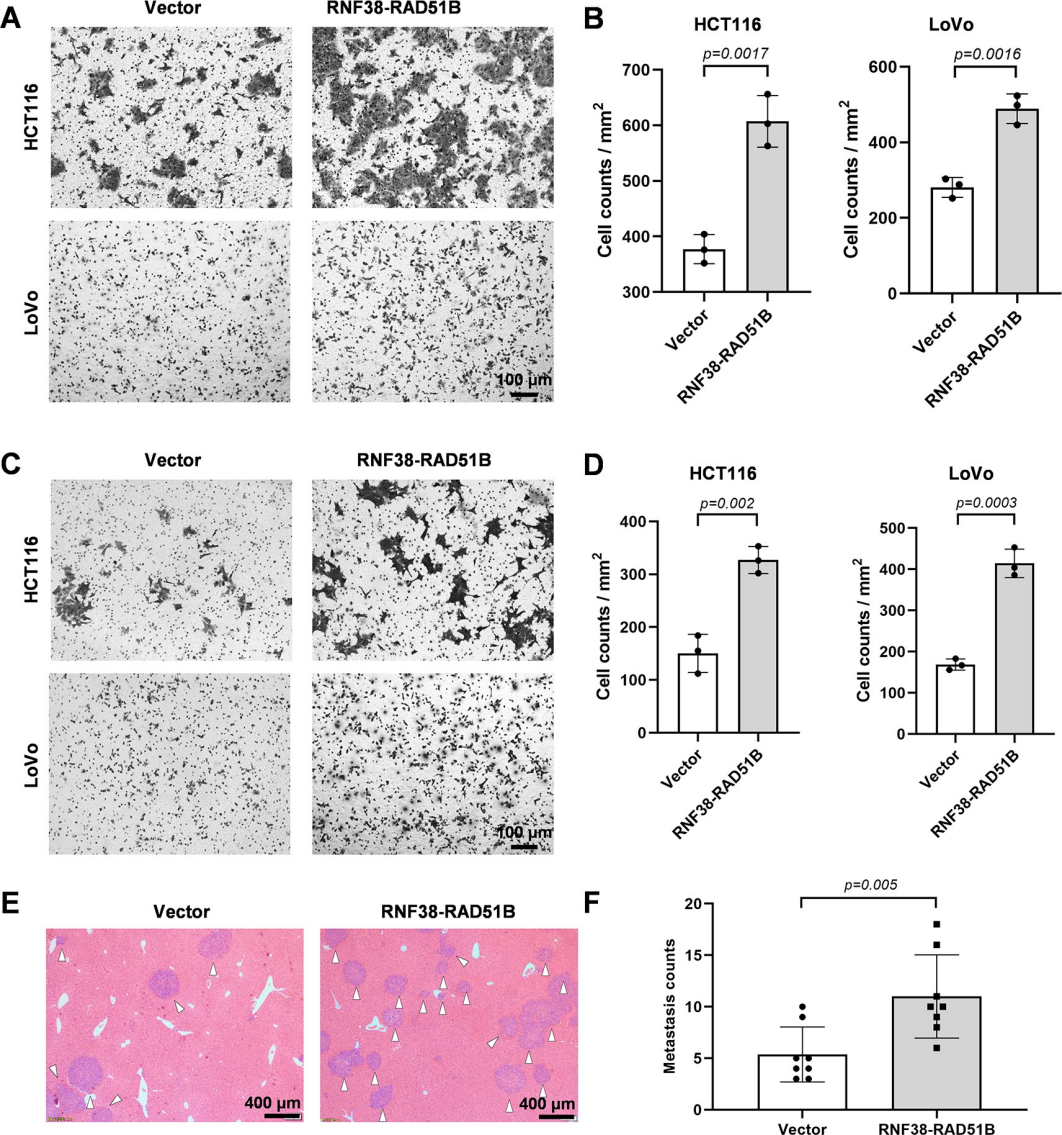
RNF38-RAD51B promotes cell migration, invasion and CRC metastasis. **(A, B)** Representative images (A) and statistical results (B) of transwell migration assay of RNF38-RAD51B overexpressing CRC cells (three repetitions per group). **(C, D)** Representative images (C) and statistical results (D) of transwell invasion assay of RNF38-RAD51B overexpressing CRC cells (three repetitions per group). **(E, F)** Representative H&E-staining images (E) and counts (F) of metastatic tumors in the liver of the xenograft mice intravenously injected with RNF38-RAD51B overexpressing HCT116 cells (eight mice per group). p < 0.05 are statistically significant (students’ *t*-test).

To investigate the oncogenic effects of the RNF38-RAD51B fusion, we cloned the fusion gene and established RNF38-RAD51B overexpressing LoVo (human colon adenocarcinoma cell line) and HCT116 (human rectum adenocarcinoma cell line) cells (S12 Fig). The overexpression of RNF38-RAD51B significantly promoted cell migration and invasion *in vitro* in transwell assays (Fig 5A–5D). We next examined the *in vivo* oncogenic roles of the RNF38-RAD51B fusion by intravenously injecting RNF38-RAD51B overexpressing HCT116 cells into nude mice. The metastasis of tumor cells into the livers was observed (Fig 5E and 5F); the number of metastatic loci were two-time higher than that in the control (injected with empty-vector expressing cells). These results demonstrate that RNF38-RAD51B fusion enhances CRC cells’ ability of migration, invasion and metastasis.

## Discussion

Structural variations are deemed as oncogenic organizers that alter expression and function of oncogenes or tumor suppressors [5]. However, due to the short read length caused ambiguous alignment, commonly used short-read sequencing strategies are ineffective in breakpoints phasing, and complex or long SVs detection and reconstruction [34] Yet, large amount of hidden structural variations in human genomes need to be further identified [15,35]. In this study, we applied nanopore long-read sequencing in 21 pairs of CRC samples, detected approximately twice numbers of somatic SVs in each sample than using short-read sequencing [24,25], and many of them were related to known oncogenes and tumor suppressors. We further investigated the types and components of SVs in CRC, and identified multiple SV hotspots that were associated with CRC-associated genes. This is the first study that employed long-read sequencing to investigate SVs in human CRC samples.

The majority of clinically-used precision therapeutics approaches for colorectal cancer, such as food and drug administration (FDA) approved MSK-IMPACT (Memorial Sloan Kettering Cancer Center) and FoundationOne CDx (Foundation Medicine, Inc) tests, used short-read capture sequencing or amplicon sequencing to detect cancer-relevant and/ or drug-targetable mutations as treatment indicators [36,37]. However, patients might not benefit from short-read capture sequencing or amplicon sequencing if their treatment indicators are SVs [38] since SVs (especially large-scale SVs) may span over one or more exons without any change in their sequence, it is highly possible that these exon-spanning SVs would be missed if using capture sequencing or amplicon sequencing. For instance, the inversions in *APC* and *CFTR* clearly altered the structure (including coding regions) of both genes, but were not detected by WES. Thus, detection of such SVs would be valuable to cancer precision therapeutics. Compared to short-read capture sequencing, long reads sequencing are advantageous in capturing large, complex SVs, and SVs in repetitive regions, as long reads (> 5 kbp) can easily span repetitive sequences or SV breakpoints, and aligned precisely [22]. In the current study, the reads spanning the 11.2-kbp inversion in *CFTR* showed that the enhanced read length enables a full capture of SVs, significantly improving cancer SVs detection efficacy, providing a powerful tool for cancer precision therapeutics.

Gene fusions resulting from genomic rearrangements, represent an important part of tumor genomic landscape and are involved in development of approximately 16% of all cancer types, including CRC [39]. Although short-reads based whole genome sequencing (WGS) and RNA-seq are two major methods for identifying fusion genes, WGS is limited by the disadvantages mentioned above, and RNA-seq suffers from poor sensitivity for detecting the fusion genes that are expressed at rather low levels or diluted by accompanying non-cancerous cells [40]. In contrast, the advantages of long-read sequencing allow more effective identification of novel genetic rearrangements that may result in gene fusions. Indeed, our work uncovered a novel gene fusion, *RNF38-RAD51B*, which could enhance CRC cells’ oncogenic functions. RNF38 was reported as a vital driver of cancer progression and could promote the invasion and metastasis of cancer cells [30,31]. The *RNF38-RAD51B* gene fusion may enhance the expression or function of RNF38, since it significantly promoted the invasion and metastasis ability of colorectal cancer cells. Although the molecular mechanisms and clinical relevance of this gene fusion need to be further studied, our results suggest that nanopore sequencing may serve as a new strategy for detecting oncogenic gene fusions.

Nevertheless, this study has some limitations. First, the sample size (21 pairs of samples) was limited, making it difficult to find low-frequency somatic SVs in CRC. Second, a higher sequencing depth would be needed to improve the accuracy of SV phasing, especially for small insertions and deletions. Third, functional studies were required for further revealing functional roles of our newly-discovered somatic SVs, even though they were likely to promote development and progression of CRC according to their impact on genes structures (i.e., the inversions altered tumor suppressors *APC* and *CFTR*).

In summary, our study provides an example illustrating the utility of long-read nanopore sequencing in cancer genome investigation. Our work highlights the potential of the long-read sequencing in serving as a new platform for the precise diagnosis and treatment of CRC, and portrayed the first landscape of somatic SVs detected by long-read sequencing in CRC, which can be a useful resource for future biological and clinical studies.

## Supporting information

S1 Fig Quantification of SVs in tumor and normal samples. The X-axis represents the patient IDs (detailed information see S1 and S2 Tables)
(PDF)

S2 Fig (A) The number of detected somatic SVs in MSS and MSI-H samples. (B) The number of detected somatic SVs in different stages.
(PDF)

S3 Fig (A and B) Quantification (A) and percentages of types (B) of somatic SVs detected by long-read sequencing in each sample. Insertions were the dominated SVs in MSIH samples. (C and D) Quantification (C) and percentages of types (D) of somatic SVs detected by long-read sequencing in each sample after the exclusion of insertions in STR regions. The X-axes in each graph represent the sample IDs.
(PDF)

S4 Fig Quantification of somatic insertions located at short tandem repeat (STR) regions between MSI-H or MSS samples (p<0.0001, Student’s t-test). (PDF)

S5 Fig The length (A) and distance (B) distributions of somatic SVs.
(PDF)

S6 Fig Quantification of singleton and recurrent somatic SVs in each sample including (A) or excluding (B) insertions in STR. The X-axes in each graph represent the patient IDs.
(PDF)

S7 Fig Percentages of somatic insertions (INS), deletions (DEL), duplications (DUP), inversions (INV) and translocations. Different colors represent different recurrence number (left of the graph) within the tested tumor samples from 21 patients.
(PDF)

S8 Fig Quantification of the numbers of LINE and SINE insertions in each tumor sample. The X-axis represents the sample IDs.
(PDF)

S9 Fig Images of electrophoresis of the products from PCR validation of the breakpoints (BP) of inversions and gene fusions. (A) The 4,915 kbp inversion that affected APC in the sample C546-T. (B) The 11.2 kbp inversion that affected CFTR in the sample C564-T. (C) The RNF38-RAD41B gene fusion. (D) The SMAD3-SHISA6 gene fusion.
(PDF)

S10 Fig Sanger sequencing results demonstrate the complex breakpoint structures at the single-base resolution. (A) The 4,915 kbp inversion that affected APC in the sample C546-T. (B) The 11.2 kbp inversion that affected CFTR in the sample C564-T.
(PDF)

S11 Fig The Sanger sequencing chromatograms of the breakpoints of the RNF38-RAD51B (A) and SMAD3-SHISA6 gene fusions (B).
(PDF)

S12 Fig The western blot result of overexpressed RNF38-RAD51B fusion gene in LoVo and HCT116 cells. The fusion gene was labelled by Flag tag.
(PDF)

S1 Table Clinical properties of the CRC patients. (PDF)

S2 Table Data summary of the long-read sequencing. (PDF)

S3 Table Data summary of the short-read whole exome sequencing. (PDF)

S4 Table The numerical data underlying the graphs or summary statistics in this study. (XLSX)
