# Supplementary material for: Long-read sequencing identifies novel structural variations in colorectal cancer
Source: PLoS Genet. 2023 Feb 22;19(2):e1010514. doi: 10.1371/journal.pgen.1010514 (PMC10013895; doi:10.1371/journal.pgen.1010514)
Supplement: S1 Fig — The X-axis represents the patient IDs (detailed information see S1 and S2 Tables) (PDF) [file pgen.1010514.s001.pdf]

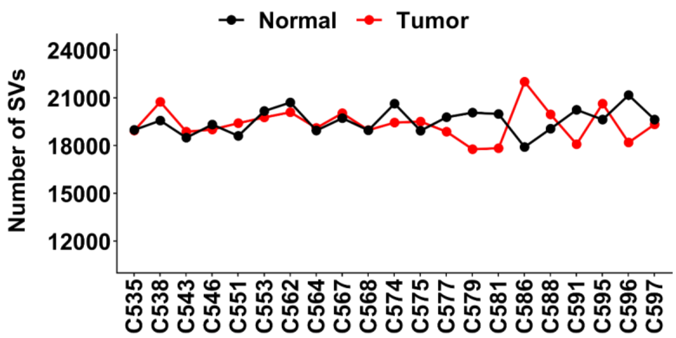

Figure S1. Quantification of SVs in tumor and normal samples. The X-axis represents the patient IDs (detailed information see Table S1 and S2).
