# Supplementary material for: Long-read sequencing identifies novel structural variations in colorectal cancer
Source: PLoS Genet. 2023 Feb 22;19(2):e1010514. doi: 10.1371/journal.pgen.1010514 (PMC10013895; doi:10.1371/journal.pgen.1010514)
Supplement: S2 Fig — (A) The number of detected somatic SVs in MSS and MSI-H samples. (B) The number of detected somatic SVs in different stages. (PDF) [file pgen.1010514.s002.pdf]

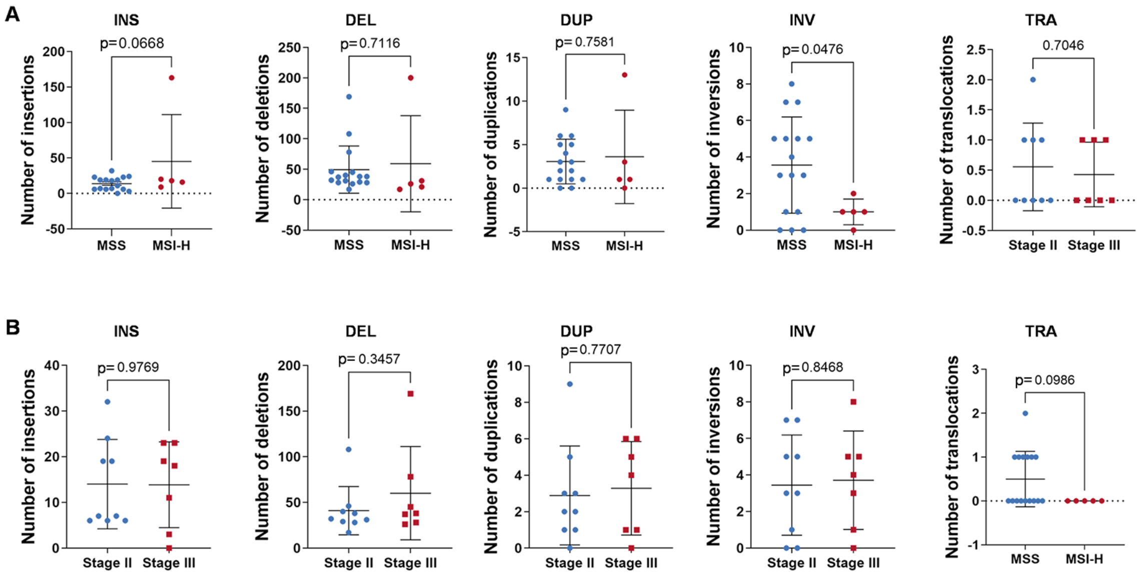

Figure S2. (A) The number of detected somatic SVs in MSS and MSI-H samples. **(B)** The number of detected somatic SVs in different stages.
