# Supplementary material for: Long-read sequencing identifies novel structural variations in colorectal cancer
Source: PLoS Genet. 2023 Feb 22;19(2):e1010514. doi: 10.1371/journal.pgen.1010514 (PMC10013895; doi:10.1371/journal.pgen.1010514)
Supplement: S3 Fig — (A and B) Quantification (A) and percentages of types (B) of somatic SVs detected by long-read sequencing in each sample. Insertions were the dominated SVs in MSIH samples. (C and D) Quantification (C) and percentages of types (D) of somatic SVs detected by long-read sequencing in each sample after the exclusion of insertions in STR regions. The X-axes in each graph represent the sample IDs. (PDF) [file pgen.1010514.s003.pdf]

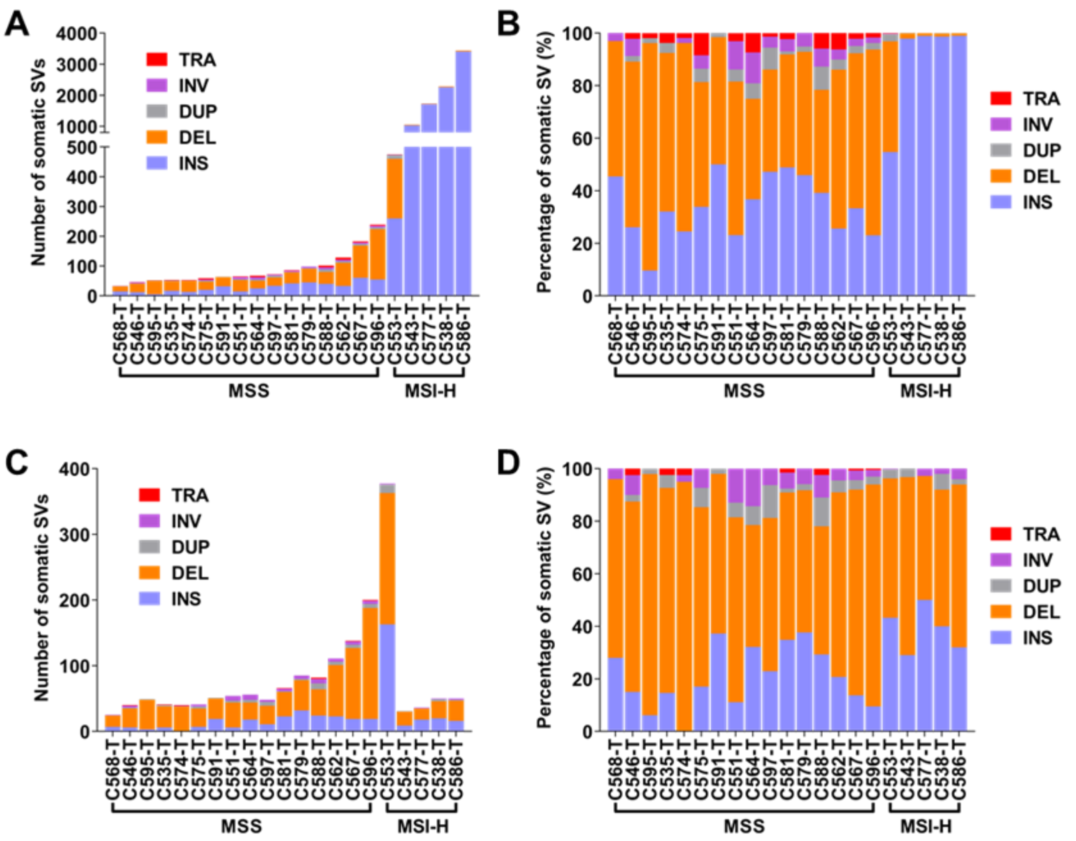

Figure S3. **(A** and **B)** Quantification (A) and percentages of types (B) of somatic SVs detected by long-read sequencing in each sample. Insertions were the dominated SVs in MSI-H samples. (**C** and **D**) Quantification (C) and percentages of types (D) of somatic SVs detected by long-read sequencing in each sample after the exclusion of insertions in STR regions. The X-axes in each graph represent the sample IDs.
