# Supplementary material for: Long-read sequencing identifies novel structural variations in colorectal cancer
Source: PLoS Genet. 2023 Feb 22;19(2):e1010514. doi: 10.1371/journal.pgen.1010514 (PMC10013895; doi:10.1371/journal.pgen.1010514)
Supplement: S4 Fig — (PDF) [file pgen.1010514.s004.pdf]

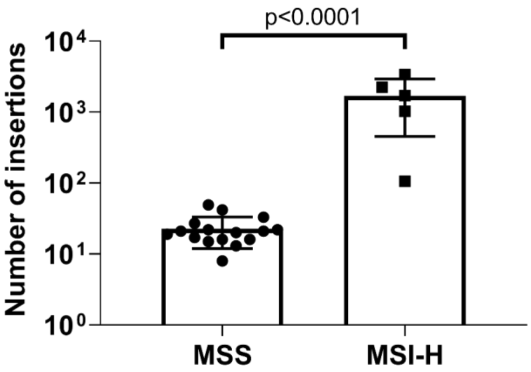

Figure S4. Quantification of somatic insertions located at short tandem repeat (STR) regions between MSI-H or MSS samples (p<0.0001, Student's *t*-test).
