# Supplementary figures and images for: Long-read sequencing identifies novel structural variations in colorectal cancer
Source: PLoS Genet. 2023 Feb 22;19(2):e1010514. doi: 10.1371/journal.pgen.1010514 (PMC10013895; doi:10.1371/journal.pgen.1010514)

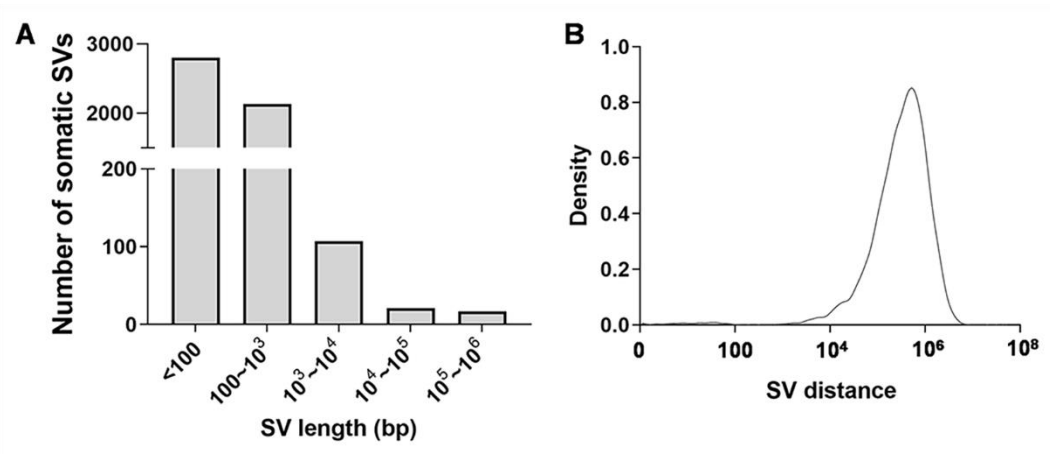

**Figure S5.** The length (**A**) and distance (**B**) distributions of somatic SVs.

Supplement: S5 Fig — The length (A) and distance (B) distributions of somatic SVs. (PDF) [file pgen.1010514.s005.pdf]
