# Supplementary material for: Long-read sequencing identifies novel structural variations in colorectal cancer
Source: PLoS Genet. 2023 Feb 22;19(2):e1010514. doi: 10.1371/journal.pgen.1010514 (PMC10013895; doi:10.1371/journal.pgen.1010514)
Supplement: S6 Fig — Quantification of singleton and recurrent somatic SVs in each sample including (A) or excluding (B) insertions in STR. The X-axes in each graph represent the patient IDs. (PDF) [file pgen.1010514.s006.pdf]

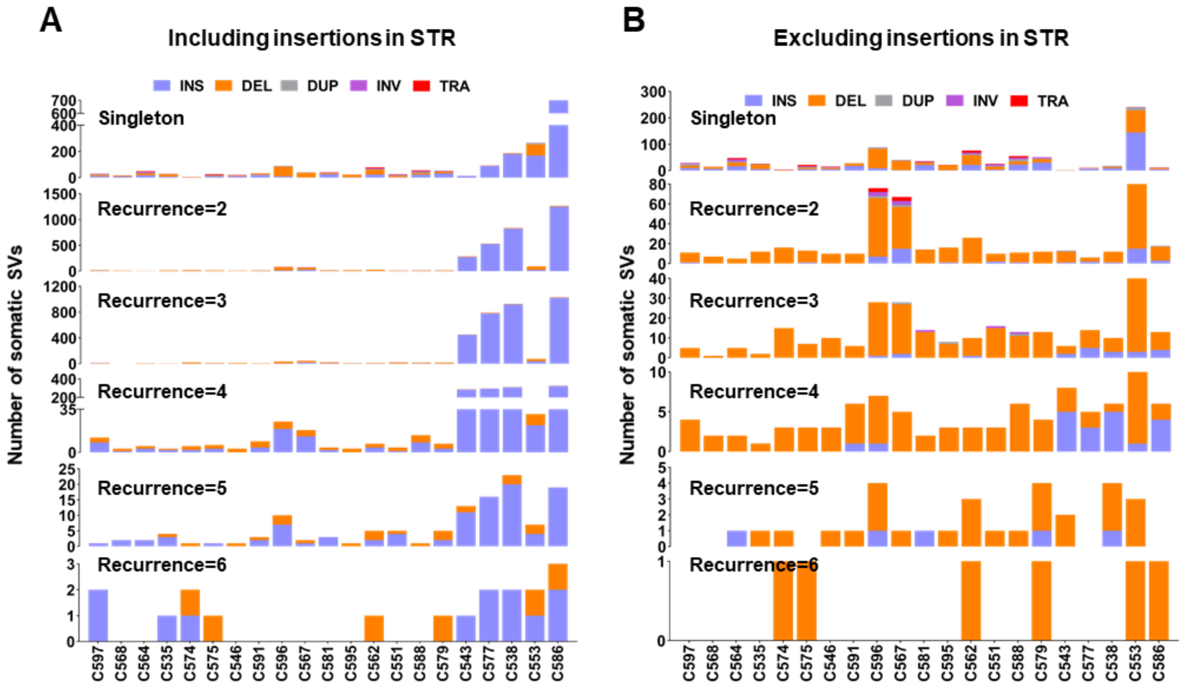

Figure S6. Quantification of singleton and recurrent somatic SVs in each sample including **(A)** or excluding **(B)** insertions in STR. The X-axes in each graph represent the patient IDs.
