# Supplementary material for: Long-read sequencing identifies novel structural variations in colorectal cancer
Source: PLoS Genet. 2023 Feb 22;19(2):e1010514. doi: 10.1371/journal.pgen.1010514 (PMC10013895; doi:10.1371/journal.pgen.1010514)
Supplement: S7 Fig — Different colors represent different recurrence number (left of the graph) within the tested tumor samples from 21 patients. (PDF) [file pgen.1010514.s007.pdf]

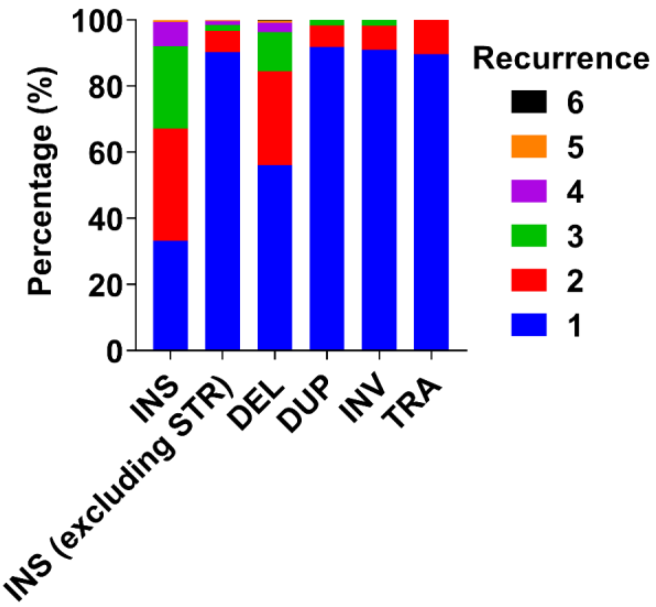

Figure S7. Percentages of somatic insertions (INS), deletions (DEL), duplications (DUP), inversions (INV) and translocations. Different colors represent different recurrence number (left of the graph) within the tested tumor samples from 21 patients.
