# Supplementary material for: Long-read sequencing identifies novel structural variations in colorectal cancer
Source: PLoS Genet. 2023 Feb 22;19(2):e1010514. doi: 10.1371/journal.pgen.1010514 (PMC10013895; doi:10.1371/journal.pgen.1010514)
Supplement: S8 Fig — The X-axis represents the sample IDs. (PDF) [file pgen.1010514.s008.pdf]

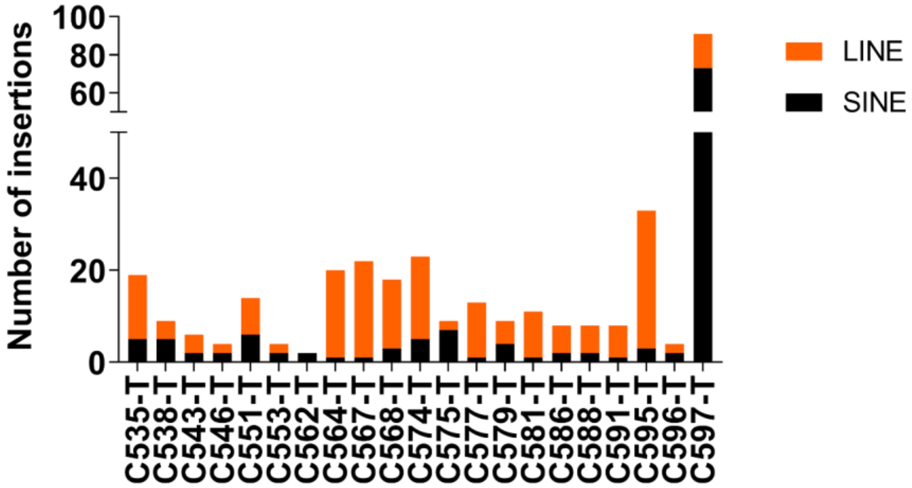

Figure S8. Quantification of the numbers of LINE and SINE insertions in each tumor sample. The X-axis represents the sample IDs.
