# Supplementary material for: Long-read sequencing identifies novel structural variations in colorectal cancer
Source: PLoS Genet. 2023 Feb 22;19(2):e1010514. doi: 10.1371/journal.pgen.1010514 (PMC10013895; doi:10.1371/journal.pgen.1010514)
Supplement: S9 Fig — (A) The 4,915 kbp inversion that affected APC in the sample C546-T. (B) The 11.2 kbp inversion that affected CFTR in the sample C564-T. (C) The RNF38-RAD41B gene fusion. (D) The SMAD3-SHISA6 gene fusion. (PDF) [file pgen.1010514.s009.pdf]

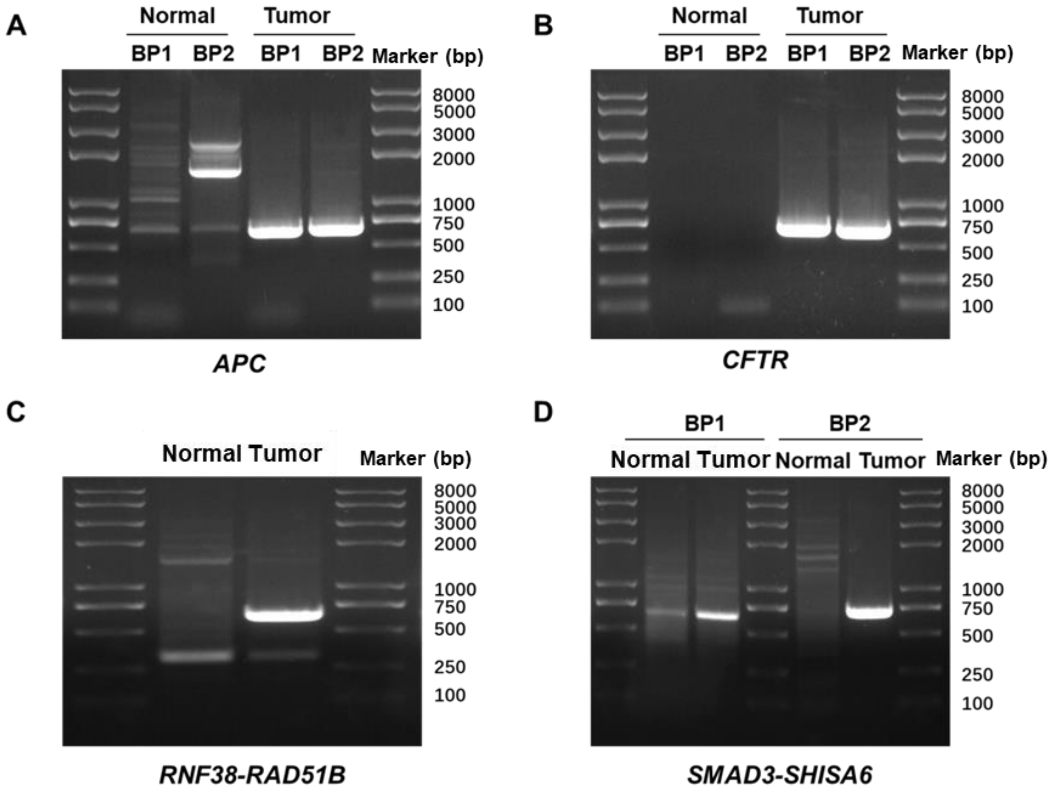

Figure S9. Images of electrophoresis of the products from PCR validation of the breakpoints (BP) of inversions and gene fusions. **(A)** The 4,915 kbp inversion that affected *APC* in the sample C546-T. **(B)** The 11.2 kbp inversion that affected *CFTR* in the sample C564-T. **(C)** The *RNF38-RAD41B* gene fusion. **(D)** The *SMAD3-SHISA6* gene fusion.
