# Supplementary material for: Long-read sequencing identifies novel structural variations in colorectal cancer
Source: PLoS Genet. 2023 Feb 22;19(2):e1010514. doi: 10.1371/journal.pgen.1010514 (PMC10013895; doi:10.1371/journal.pgen.1010514)
Supplement: S10 Fig — (A) The 4,915 kbp inversion that affected APC in the sample C546-T. (B) The 11.2 kbp inversion that affected CFTR in the sample C564-T. (PDF) [file pgen.1010514.s010.pdf]

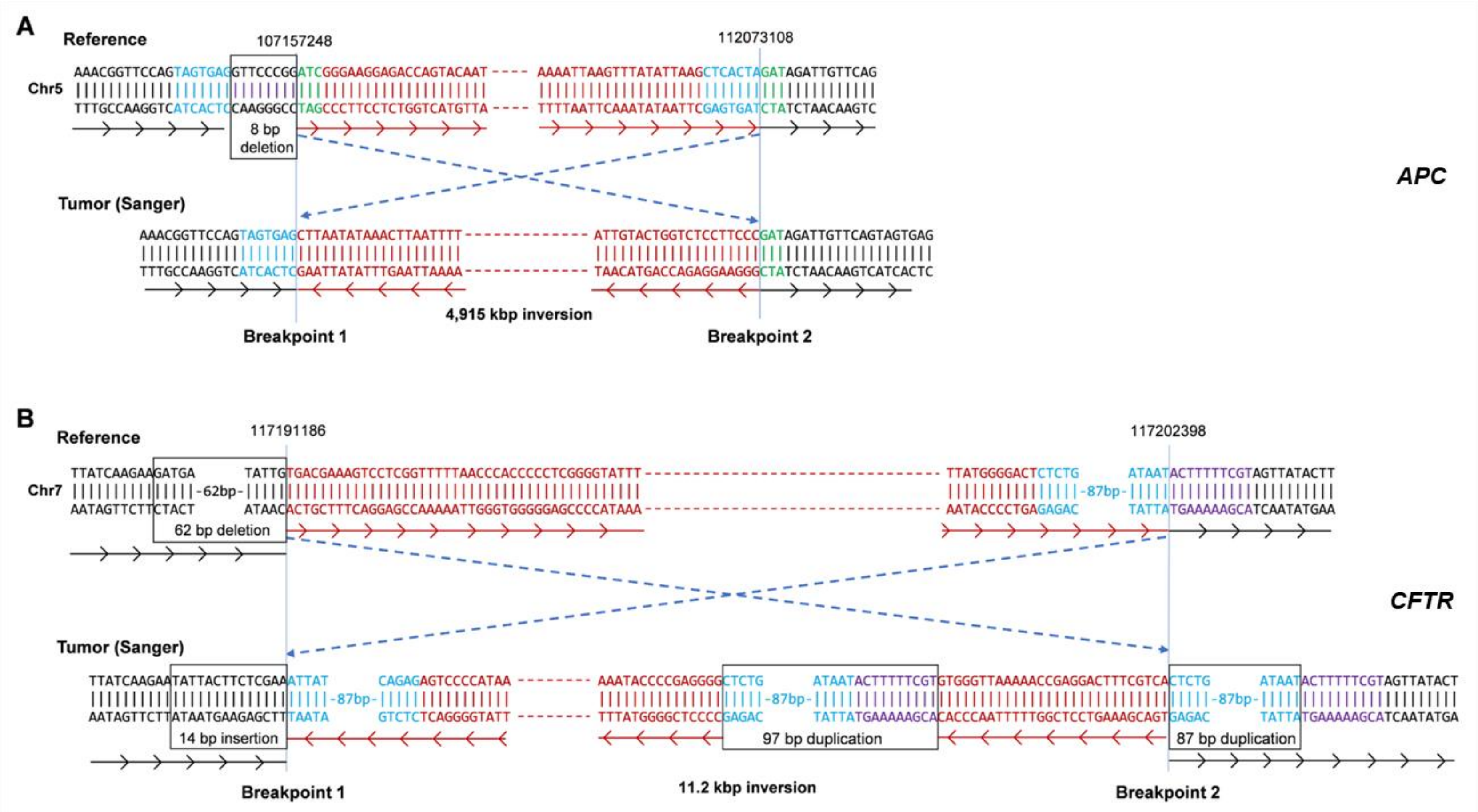

Figure S10. Sanger sequencing results demonstrate the complex breakpoint structures at the single-base resolution. **(A)** The 4,915 kbp inversion that affected *APC* in the sample C546-T. **(B)** The 11.2 kbp inversion that affected *CFTR* in the sample C564-T.
