# Supplementary material for: Long-read sequencing identifies novel structural variations in colorectal cancer
Source: PLoS Genet. 2023 Feb 22;19(2):e1010514. doi: 10.1371/journal.pgen.1010514 (PMC10013895; doi:10.1371/journal.pgen.1010514)
Supplement: S11 Fig — The Sanger sequencing chromatograms of the breakpoints of the RNF38-RAD51B (A) and SMAD3-SHISA6 gene fusions (B). (PDF) [file pgen.1010514.s011.pdf]

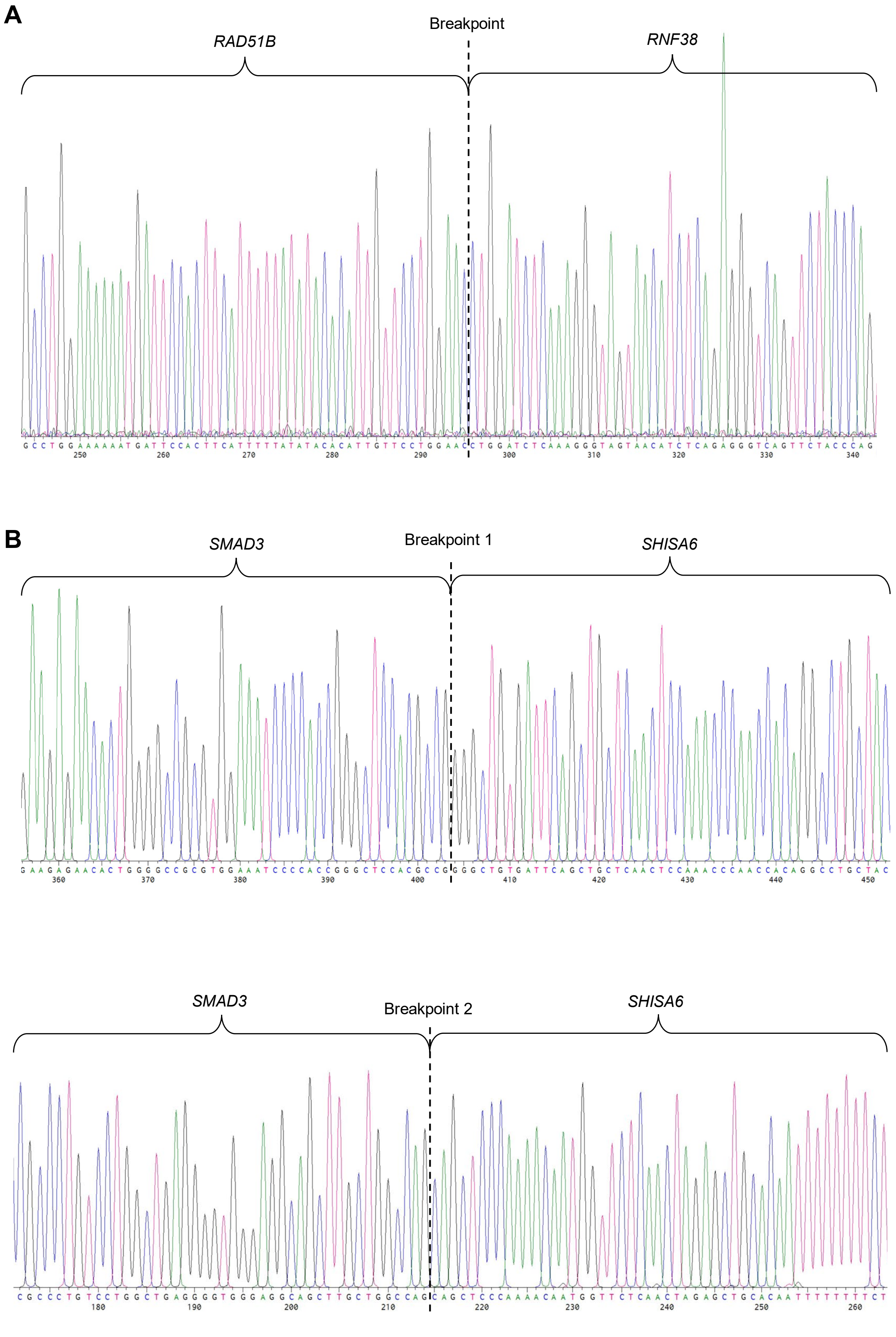

Figure S11. The Sanger sequencing chromatograms of the breakpoints of the RNF38-RAD51B **(A)** and SMAD3-SHISA6 gene fusions **(B)**.
