# Supplementary material for: Long-read sequencing identifies novel structural variations in colorectal cancer
Source: PLoS Genet. 2023 Feb 22;19(2):e1010514. doi: 10.1371/journal.pgen.1010514 (PMC10013895; doi:10.1371/journal.pgen.1010514)
Supplement: S12 Fig — The fusion gene was labelled by Flag tag. (PDF) [file pgen.1010514.s012.pdf]

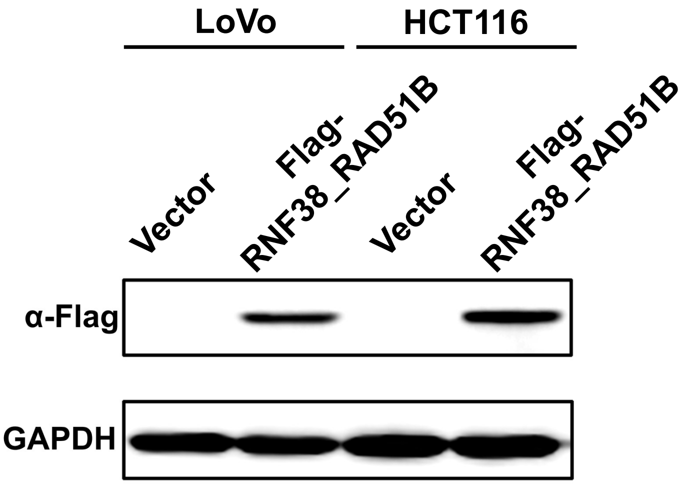

Figure S12. The western blot result of overexpressed RNF38-RAD51B fusion gene in LoVo and HCT116 cells. The fusion gene was labeled by Flag tag.
