# Supplementary material for: Long-read sequencing identifies novel structural variations in colorectal cancer
Source: PLoS Genet. 2023 Feb 22;19(2):e1010514. doi: 10.1371/journal.pgen.1010514 (PMC10013895; doi:10.1371/journal.pgen.1010514)
Supplement: S1 Table — (PDF) [file pgen.1010514.s013.pdf]

Table S1. Clinical properties of the CRC patients

**Patient ID**	**Population**	**Gender**	**Age**	**Tumor site**	**Pathological type**	**TNM stage at diagnosis**	**MSI status**	**Prior therapy**
C535	Chinese	Female	73	Sigmoid colon	Adenocarcinoma	II	MSS	No
C538	Chinese	Female	76	Ascending colon	Adenocarcinoma	III	MSI-H	No
C543	Chinese	Male	55	Ascending colon	Adenocarcinoma	II	MSI-H	No
C546	Chinese	Female	29	Rectum	Adenocarcinoma	II	MSS	No
C551	Chinese	Female	69	Rectum	Adenocarcinoma	II	MSS	No
C553	Chinese	Female	65	Ascending colon	Adenocarcinoma	II	MSI-H	No
C562	Chinese	Male	63	Rectum	Adenocarcinoma	III	MSS	No
C564	Chinese	Female	67	Ascending colon	Adenocarcinoma	III	MSS	No
C567	Chinese	Female	62	Rectum	Adenocarcinoma	II	MSS	No
C568	Chinese	Male	55	Rectum	Adenocarcinoma	II	MSS	No
C574	Chinese	Male	62	Rectum	Adenocarcinoma	III	MSS	No
C575	Chinese	Female	55	Rectum	Adenocarcinoma	II	MSS	No
C577	Chinese	Female	61	Ascending colon	Adenocarcinoma	II	MSI-H	No
C579	Chinese	Male	79	Ascending colon	Adenocarcinoma	II	MSS	No
C581	Chinese	Male	62	Sigmoid colon	Adenocarcinoma	III	MSS	No
C586	Chinese	Female	53	Ascending colon	Adenocarcinoma	II	MSI-H	No
C588	Chinese	Male	66	Ascending colon	Adenocarcinoma	II	MSS	No
C591	Chinese	Male	63	Rectal/ sigmoid junction	Adenocarcinoma	II	MSS	No
C595	Chinese	Male	56	Descending colon	Adenocarcinoma	III	MSS	No
C596	Chinese	Female	44	Splenic flexure	Adenocarcinoma	III	MSS	No
C597	Chinese	Female	67	Rectum	Adenocarcinoma	III	MSS	No
