# Supplementary material for: Long-read sequencing identifies novel structural variations in colorectal cancer
Source: PLoS Genet. 2023 Feb 22;19(2):e1010514. doi: 10.1371/journal.pgen.1010514 (PMC10013895; doi:10.1371/journal.pgen.1010514)
Supplement: S2 Table — (PDF) [file pgen.1010514.s014.pdf]

Table S2. Data summary of the long-read sequencing

Sample	Total bases	Reads counts	Mean read length (bp)	Max read length (bp)	N50 (bp)	Depth (X)
C535-N	57,669,364,676	2,898,375	19,897	345,807	28,766	19.22
C535-T	57,312,064,766	3,195,333	17,936	637,187	23,714	19.10
C538-N	61,051,050,744	3,325,985	18,355	607,177	26,313	20.35
C538-T	61,631,579,244	3,638,574	16,938	541,366	24,052	20.54
C543-N	67,602,529,867	3,550,109	19,042	378,399	29,961	22.53
C543-T	67,281,259,538	3,729,894	18,038	497,071	28,006	22.42
C546-N	48,774,891,427	3,421,925	14,253	886,832	19,238	16.26
C546-T	60,881,983,808	3,592,313	16,947	352,520	24,218	20.29
C551-N	37,714,254,433	1,423,112	26,501	337,466	40,465	12.57
C551-T	51,977,018,011	1,924,112	27,013	354,789	41,724	17.32
C553-N	64,244,629,656	2,843,441	22,593	1,114,499	33,701	21.41
C553-T	82,460,268,022	4,546,175	18,138	244,312	22,213	27.48
C562-N	42,724,960,442	2,360,794	18,097	339,453	25,216	14.24
C562-T	42,568,118,644	2,110,128	20,173	409,553	31,961	14.19
C564-N	56,357,599,210	2,680,607	21,024	316,921	28,817	18.78
C564-T	49,451,863,348	3,258,078	15,178	395,922	22,920	16.48
C567-N	50,487,298,586	2,861,899	17,641	371,189	28,287	16.83
C567-T	28,068,765,306	1,400,952	20,035	353,634	30,320	9.35
C568-N	56,554,996,338	2,333,221	24,239	270,467	35,221	18.85
C568-T	48,550,544,375	2,697,254	17,999	285,010	22,275	16.18
C574-N	43,689,527,345	1,561,013	27,987	374,466	45,166	14.56
C574-T	73,820,480,665	3,002,383	24,587	361,794	40,862	24.60
C575-N	57,740,041,362	2,566,683	22,495	559,555	32,753	19.24
C575-T	64,742,630,530	3,507,195	18,459	558,527	27,274	21.58
C577-N	40,351,153,094	1,744,309	23,133	475,556	37,558	13.45
C577-T	55,604,752,045	3,629,356	15,320	454,025	23,495	18.53
C579-N	40,959,163,844	2,279,386	17,969	353,671	27,001	13.65
C579-T	57,865,143,193	2,432,274	23,790	408,970	39,403	19.29
C581-N	39,743,869,056	1,730,317	22,969	751,107	40,350	13.25

C581-T	46,903,319,537	2,062,119	22,745	407,302	39,106	15.63
C586-N	45,291,368,283	2,918,315	15,519	679,447	21,435	15.09
C586-T	57,147,936,154	2,624,259	21,776	708,834	34,799	19.05
C588-N	37,366,467,378	1,877,006	19,907	367,823	28,373	12.45
C588-T	54,435,049,311	3,447,597	15,789	455,050	25,509	18.14
C591-N	54,723,348,149	2,547,666	21,479	432,306	34,176	18.24
C591-T	35,386,368,343	1,362,750	25,966	466,810	42,969	11.79
C595-N	50,414,592,294	2,997,882	16,816	798,307	22,119	16.80
C595-T	43,542,968,780	1,973,038	22,068	897,996	34,921	14.51
C596-N	44,708,429,781	2,754,851	16,228	354,365	22,426	14.90
C596-T	28,703,982,345	2,003,932	14,323	432,447	21,361	9.57
