# Supplementary material for: Long-read sequencing identifies novel structural variations in colorectal cancer
Source: PLoS Genet. 2023 Feb 22;19(2):e1010514. doi: 10.1371/journal.pgen.1010514 (PMC10013895; doi:10.1371/journal.pgen.1010514)
Supplement: S3 Table — (PDF) [file pgen.1010514.s015.pdf]

Table S3. Data summary of the short-read whole exome sequencing

Sample	Total bases (G)	Q20 bases (G)	Q30 bases (G)	Depth (X)
C535-N	10.561365	10.24401	10.039725	203.1032
C535-T	12.691394	12.398178	11.920065	244.0653
C538-N	12.173638	11.903818	11.48697	234.1084
C538-T	13.187507	12.935168	12.496216	253.6059
C543-N	12.996943	12.764905	12.358564	249.9412
C543-T	12.621266	12.397011	12.003054	242.7167
C546-N	14.093436	13.768507	13.269046	271.0276
C546-T	12.677241	12.457216	12.068948	243.7931
C551-N	12.499303	12.34212	12.05733	240.3712
C551-T	12.873985	12.71013	12.415078	247.5766
C553-N	13.244775	13.060263	12.735929	254.7072
C553-T	13.076255	12.89722	12.579244	251.4664
C562-N	15.675952	15.459685	15.077405	301.4606
C562-T	15.107745	14.873153	14.470456	290.5336
C564-N	12.026852	11.851629	11.547349	231.2856
C564-T	13.072649	12.88805	12.565057	251.3971
C567-N	14.180633	13.982639	13.634757	272.7045
C567-T	13.526872	13.338547	13.006695	260.1322
C568-N	12.187114	12.01234	11.706836	234.3676
C568-T	14.143086	13.932977	13.564625	271.9824
C574-N	11.971365	11.81437	11.544378	230.2186
C574-T	8.703624	8.601943	8.422413	167.3774
C575-N	12.541381	12.401152	12.149901	241.1804
C575-T	14.523402	14.365019	14.07919	279.2962
C577-N	15.06086	14.88451	14.573771	289.6319
C577-T	15.415049	15.231944	14.907402	296.4433
C579-N	15.391165	15.211476	14.893351	295.9839
C579-T	15.453413	15.265212	14.934961	297.181
C581-N	14.11636	13.94418	13.641807	271.4685
C581-T	16.058453	15.864686	15.524244	308.8164
C586-N	14.084198	13.910763	13.606044	270.85
C586-T	17.190521	16.976111	16.59587	330.5869
C588-N	15.66323	15.482512	15.157711	301.216
C588-T	17.6349	17.427615	17.055364	339.1327
C591-N	11.609484	11.345355	10.905893	223.2593
C591-T	16.203788	15.999961	15.639406	311.6113
C595-N	17.438429	17.228802	16.854687	335.3544
C595-T	15.634228	15.28226	14.706422	300.6582
C596-N	4.670272	4.59657	4.476274	89.81292
C596-T	15.999868	15.741545	15.363514	307.6898
C597-N	15.96157	15.772334	15.434657	306.9533

C597-T	13.817309	13.650313	13.352744	265.7175
