# Supplementary material for: Long-read sequencing identifies novel structural variations in colorectal cancer
Source: PLoS Genet. 2023 Feb 22;19(2):e1010514. doi: 10.1371/journal.pgen.1010514 (PMC10013895; doi:10.1371/journal.pgen.1010514)
Supplement: Attachment — Submitted filename: Response to the reviewers.docx [file pgen.1010514.s017.docx]

*Union Hospital, Tongji Medical College, Huazhong University of Science and Technology,*

*1277 Jiefang Avenue, Wuhan, China 430022*

Dear *Editor*,

We thank you very much for your and reviewer’s constructive comments and suggestions on our manuscript entitled “*Long-read sequencing identifies novel structural variations in colorectal cancer*”. We have carefully studied the comments and suggestions, and have revised the manuscript accordingly. Revised portions are highlighted in red in the manuscript. We would like to express our sincere appreciation to you and reviewers for comments and suggestions on our manuscript.

Your sincerely,

**Zheng Wang, MD/ PhD**

*Professor of Medicine,*

*Department of Gastrointestinal Surgery,*

*Research Center for Tissue Engineering and Regenerative Medicine,*

*Union Hospital, Tongji Medical College,*

*Huazhong University of Science and Technology*

*1277 Jiefang Ave., Wuhan, China 430022*

*zhengwang@hust.edu.cn*

*Tel: +86-27-85726612*

**Lin Wang, PhD**

*Professor and Director,*

*Research Center for Tissue Engineering and Regenerative Medicine,*

*Department of Clinical Laboratory,*

*Union Hospital, Tongji Medical College,*

*Huazhong University of Science and Technology*

*1277 Jiefang Ave., Wuhan, China 430022*

*lin_wang@hust.edu.cn*

*Tel: +86-27-85726612*

**Journal**: Genome Medicine

**Manuscript ID**: PGENETICS-D-21-01626

**Title**: Long-read sequencing identifies novel structural variations in colorectal cancer

**Responses to Reviewers’ Comments**

*Thank the reviewers very much for the comments and suggestions that greatly help make this manuscript much better!*

**Reviewer #1**: The 'Long-read sequencing identifies novel structural variations in colorectal cancer' describes the assessment of long read sequencing (ONT) as a method to investigate somatic SVs in 21 colorectal cancer samples. The data presented has useful insights into the use of ONT for the detection of somatic SVs. However, many of the methods and results lack sufficient details and make it difficult to interpret the findings. While I believe the data generated for the manuscript has merit and would be of interest to the community, I think a significant amount of additional information describing the data and revisions to the manuscript to improve its clarity are required before it is suitable for publication.

I would highly recommend English editing for the manuscript to improve grammar and clarity. While the manuscript is readable there are sentences and paragraphs that are especially unclear that make interpretation of results difficult.

Introduction

• I think the claim that short read sequencing has high false positive rates should be softened or justified as some callers have been shown to have high precision.

*We thank the reviewer very much for the comment. We have changed the corresponding text to “…and short-read sequencing is inefficient in detecting long, complex, or repetitive-region located SVs” at the first paragraph of “Introduction” section on Page 3.*

• The final paragraph highlighting the findings is particularly hard to follow.

*We thank the reviewer very much for the comment. We have carefully revised the introduction section and added the text “Long-read sequencing technologies can generate long continuous reads (length over tens of kilobase pairs (kbp)), possess increased reliability and sensitivity in SVs detection. Pacific Bioscience (as called single-molecule, read-time (SMAT) sequencing or PacBio sequencing) and Oxford Nanopore Technologies (ONT, or nanopore sequencing) are the two major strategies of long-read sequencing. Unlike short-read sequencing, PacBio and nanopore sequencing generate reads directly from native DNA (without ultrasonic / enzymatic fragmentation and PCR amplification), avoid the difficulty of detecting variants in genome regions with repeat content or atypical GC content. The advantages of long-read sequencing in studying human diseases were highlighted in several studies. For instance, a pentanucleotide repeat expansion in SAMD12 that may cause familial cortical myoclonic tremor with epilepsy was identified using nanopore sequencing. This type of repetitive-region residing SVs were difficult to be analyzed by short-read sequencers. In addition to this, long read sequencing identified the leukoencephalopathy-related GGC repeat expansions, X-linked Dystonia-Parkinsonism-related SINE-VNTR-Alu retrotransposon insertions, and these variants were previously missed by short read sequencing. Moreover, long read sequencing realized fast and low-cost genome sequencing of pathogen, such as SARS-CoV-2.” to the second paragraph of the “introduction” section on Page 3, and “showed the representative large-scale inversions that…” at the final paragraph of the “introduction” section on Page 4.*

Methods
• It is unclear how the manual curation of SVs was conducted and what was specifically was being curated.

*We thank the reviewer very much for the comment. The somatic SVs were manual curated by manually examining the reads alignment images generated from svhawkeyes (https://github.com/yywan0913/SVhawkeye). Somatic SVs that appear in both cancer and paired normal samples were identified as false-positive. We have added the text “We used svhawkeyes (https://github.com/yywan0913/SVhawkeye) for the manual curation of unfiltered somatic SVs. The reads alignment images of each unfiltered somatic SVs were generated by svhawkeyes from alignment files and manually checked. Somatic SVs that appear in both cancer and paired normal samples were identified as false positive.” at the “Alignment and SV calling” section on Page 5.*

• It is unclear if and why different reference genomes were used.

*We thank the reviewer very much for pointing this mistake out. The reference genome used for the NGMLR alignment was from NCBI (ftp://ftp-trace.ncbi.nih.gov/1000 genomes/ftp/technical/reference/human_g1k_v37.fasta.gz). We have changed the corresponding text to “…reference genome (humanG1Kv37) with …” at the “Nanopore sequencing of CRC samples” section on Page 8.*

• How variants were annotated is unclear. Databases should include version numbers.

*We thank the reviewer very much for the comments. All the somatic SVs were annotated in the segdup (UCSC golden path hg19), rmsk (UCSC golden path hg19), dgv (2016-05-15), 1000 Genome Project (phase 3), gnomAD (2.1.1), and COSMIC databases (v70) using ANNOVAR (2017-07-17) software. Besides, Insertions were further annotated as tandem repeats or known repeat classes using TRF (4.09) and RepeatMasker (4.1.0). We have added the text “SVs with upstream and downstream genes were annotated in the segdup (UCSC golden path hg19), rmsk (UCSC golden path hg19), dgv (2016-05-15), 1000 Genome Project (phase 3), gnomAD (2.1.1), and COSMIC databases (v70) using ANNOVAR (2017-07-17). Insertions were further annotated as tandem repeats or known repeat classes using TRF (4.09) and RepeatMasker (4.1).” to the “Alignment and SVs calling” section on Page 5, and the text “All somatic mutations and Indels were annotated in the dbSNP 147, clinvar (2017-05-01), ExAC (2016-04-23), 1000 Genome Project (phase 3), gnomAD (2.1.1), InterVar (2017-02-02) and COSMIC databases (v70) using ANNOVAR (2017-07-17).” to the “Whole exome sequencing and variants calling” section on Page 6.*

• The information specific to the in vitro work is severely lacking. I would have expected to see basic information describing cell culture and experimental conditions would be included (E.g. Where cells came from, growth conditions, media…) as well as more experimental information (E.g. how many replicates per experiment). More details about the mice and the tumours would also benefit the manuscript.

*We thank the reviewer very much for the comments. We have added the details of cell lines and the establishment of RNF38-RAD51B overexpressing cell lines to the “Method” section on Page 7, and added the texts “Each experiment was repeated for thrice.” and “…eight mice per group…” to the “Transwell migration and invasion assays” and “Animal studies” sections at Page 8. The replicates of each experiment were also mentioned in the legend of Figure 5.*

Results
• It is unclear if all samples were sequenced and analysed with each different technology (Short read DNA and RNA and ONT?). This should also be addressed in the methods.

*We thank the reviewer very much for the comments. All the 21 pairs tumor samples and matched normal samples were sequenced and analyzed using short-read whole exome sequencing, RNA sequencing, and long-read Nanopore sequencing. We have added the text “All the samples were analyzed and sequenced using long-read Nanopore sequencing, short-read whole exome sequencing and RNA sequencing.” to the “Sample collection and Oxford nanopore sequencing” section on Page 4, and added the text “All samples were also analyzed by short-read whole exome sequencing (WES) and RNA-seq to obtain SNVs and gene transcriptional data, respectively.” at the “Nanopore sequencing of CRC samples” section on Page 8.*

• Details describing the short DNA or RNA sequencing need to be included. There is no mention of sequencing depth or quality for either and no comparison between is provided. E.g. if SVs were also called by short read sequencing, what is the overlap?

*We thank the reviewer very much for the comments. The depth of short-read whole exome sequencing (WES) was 200×, and the data size of RNA-seq was at least 6 G. Since WES (not WGS) was unsuitable for SV detection, we did not perform the comparisons of SV calling between short and long read sequencing.*

*We have added the text “The sequencing depth of each sample was above 200X.” at the “Whole exome sequencing and variants calling” section on Page 6, and “…and at least 6 G of clean data were generated for each sample.” at the “Transcriptome sequencing and quantification of gene expression level” section on Page 6. The summary of WES data was also added to the revised manuscript as new table S3.*

• Addition of genome wide methylation results/findings would benefit the manuscript.

*We thank the reviewer very much for this comment. We performed whole genome methylation analysis for all the tumor and paired normal samples, and an obvious hypomethylation of tumor samples was observed (showed as Figure R1), but it is as expected and is well known. Since the reliability of 5-mC caller (Nanopolish) needs to be further verified, we have removed the methylation results in the revised manuscript. The detection of 5-mC via nanopore sequencing will be investigated in our further studies.*

*Figure R1. The whole genome CpG island methylation level of the samples*

• I would have liked to see a breakdown of variants selected for and filtered out for a paper that is evaluating ONT SV calling. E.g. how many variants were filtered based on the different criteria.

*We thank the reviewer very much for the comments. Somatic SVs (present in tumor but not in normal samples SV calls) were obtained by comparing high quality tumor samples SVs (supported by at least 0.3 folds of average sequencing depth) with normal samples SVs (supported by two or more reads). We obtained ~20,000 SVs in each tumor samples, and 14508 candidate somatic SVs initially. All somatic SVs were manual curated, and then 5200 SVs were left. We have added the text “…yielding 14508 unfiltered somatic SVs. After manual curation (see Method), we obtained 494 somatic SVs…” at the “Long-read sequencing identified widespread somatic SVs in colorectal cancer” section on Page 8.*

• The SVs are described as high confidence however no evidence or justification of this given.

*We thank the reviewer very much for the comment. To ensure the reliability of detected somatic SVs, we manually checked the alignment images (generated by svhawkeyes) of all the 14508 unfiltered somatic SVs. Only SVs that met the following requirements were considered as high-confidence somatic SVs: (1) supported by at least 0.3 fold of average sequencing depth; (2) only detected in tumor samples. We have added the details of the manually checking process to the “Alignment and SVs calling” section on Page 5.*

• If the over expressing cell lines were created, details about this should be included and characteristics of these cells should be described (E.g. what vector, what level of overexpression). This also applies to methods.

*We thank the reviewer very much for the comment. The vector we used for the establishment of RNF38-RAD51B overexpressing cells was pLenti-puro, and the expression level of RNF38-RAD51B fusion gene was confirmed by western blot. We have added the western blot results of RNF38-RAD51B fusion gene to the revised manuscript as new Figure S10, and added the details of the establishment of RNF38-RAD51B fusion gene overexpressing cells into the “Method” section.*

• I think the finding of the fusion event and methylation patterns is interesting, but it should be highlighted as an example finding, given that no other events were reported for the majority of the 21 cases.

*We thank the reviewer very much for the comment. We have added the text “Long read sequencing have proven immensely helpful in detecting gene fusions. For instance…” to the “Novel gene fusions identified by long-read sequencing” section on Page 10.*

Discussion
• The discussion is reasonably fair but would benefit from English editing.

*We thank the reviewer very much for the comment. We have carefully revised the discussion section.*

Other comments
1. Primer sequences should be included and I would have liked to see Sanger chromatograms in supplementary to show breakpoints of the fusion.

*We thank the reviewer very much for the comment. We have added the primer sequences for the detection of inversions (APC and CFTR) and gene fusions (RNF38-RAD51B and SMAD3-SHISA6) into the “Method” section on Page 6, and the chromatograms of sanger sequencing of RNF38-RAD51B and SMAD3-SHISA6 gene fusions were in new Figure S9.*

**Reviewer #2**: This is a study that uses long-read sequencing to identify structural variants from colorectal cancer (CRC). The study used 21 pairs of CRC samples on Nanopore whole-genome long-read sequencing which is an impressive sample size for this type of studies. In addition to novel structural variants, they also identified two novel gene fusions that may be of clinical significance. Overall this is a unique study with relatively large sample size and appropriate functional validation, further demonstrating the power of long-read sequencing in the understanding of the human genome. My comments are below to help improve the manuscript:

1, The introduction section needs expansion to give appropriate background to readers who are not familiar with long-read sequencing. In particular, several different techniques for long-read sequencing need to be summarized, before focusing on Nanopore sequencing. Additionally, quite a number of manuscripts are published over the past few years illustrating the power of long-read (some used Nanopore) sequencing to identify likely pathogenic SVs that are missed by short-read sequencing, and I think these studies should be mentioned, even though the prior studies focused on genetic diagnosis of Mendelian diseases rather than somatic driver mutations in cancer. The relative merits, including cost, speed, DNA requirements, etc should be slightly described or discussed as well. These will give readers who are not familiar with the field the necessary background information to appreciate the study.

*We thank the reviewer very much for the comment. We have added the text “Long-read sequencing technologies can generate long continuous reads (length over tens of kilobase pairs (kbp)), and possess increased reliability and sensitivity in SVs detection. Pacific Bioscience (as called single-molecule, read-time (SMAT) sequencing or PacBio sequencing) and Oxford Nanopore Technologies (ONT, or nanopore sequencing) are the two major strategies of long-read sequencing. Unlike short-read sequencing, PacBio and nanopore sequencing generate reads directly from native DNA (without ultrasonic / enzymatic fragmentation and PCR amplification), avoiding the difficulty of detecting variants in genome regions with repeat content or atypical GC content. The advantages of long-read sequencing in studying human diseases were highlighted in several studies. For instance, a pentanucleotide repeat expansion in SAMD12 that may cause familial cortical myoclonic tremor with epilepsy was identified using nanopore sequencing. This type of repetitive-region residing SVs were difficult to be analyzed by short-read sequencers. In addition to this, long-read sequencing identified the leukoencephalopathy-related GGC repeat expansions, X-linked Dystonia-Parkinsonism-related SINE-VNTR-Alu retrotransposon insertions, and these variants were previously missed by short-read sequencing. Moreover, long-read sequencing realized fast and low-cost genome sequencing of pathogens, such as SARS-CoV-2.” to the second paragraph of the “introduction” section on Page 3.*

2, it may have been mentioned elsewhere in the paper, but I cannot find a detailed description of the “21 pairs of tumor samples and matched para-carcinoma samples”. How are they ascertained, how are the samples retrieved, and are they freshly frozen? What are the pathological findings on these patients? These are important questions that need to be addressed.

*We thank the reviewer very much for the questions. All the samples in this study were collected from the surgically removed tumor tissues and adjacent intestinal tissues, and stored at -80℃. The distance between tumor and normal tissues were at least 6 cm. All tumors were pathologically diagnosed as adenocarcinoma. We have added the text “21 pairs of tumor samples and matched para-carcinoma samples were obtained from the surgically removed tumor tissues and adjacent intestinal tissues (>6 cm from tumor tissues) of CRC patients in Wuhan Union Hospital, and stored at -80℃” into the “Sample collection and Oxford nanopore sequencing” section on Page 4, and added the pathological diagnosis result to new Table S1.*

3, Since Guppy 6 is already available today, I would suggest the authors to at least basecall a couple of samples to see whether it makes a difference in terms of detecting the SVs highlighted in the paper. My guess is that the difference on SVs may be minor since the different Guppy improved basecalling accuracy for SNP/indel calling, but it would be good to see this more quantitatively.

*We thank the reviewer very much for this suggestion. We used Guppy 6.0.1 for the basecalling of two couples of samples (including the sample with CFTR inversion). Compared with Guppy 2.0.3 (88.2%), the* *average mapping identity of Guppy 6.0.1 analyzed data improved to be 97.7%. We further investigated whether Guppy 6.0.1 made a difference in SV calling. The overlap rate of SVs called from the data analyzed by Guppy 6.0.1 and 2.0.3 was ~75%, and the CFTR inversion was detected in both Guppy 6.0.1 and 2.0.3 analyzed data. These results showed that the improved basecalling accuracy of new Guppy could increases the mapping identity of ONT sequencing data, and may results in a change in the SV calling result.*

4, The study included five pairs of MSI-H samples, which as expected has abnormal expansion of short tandem repeat (STR) regions (Figure 2C). This is an interesting aspect that there is clear advantage of long-read sequencing over short-read sequencing, but I do not see much technical details on how the STR expansion is identified and quantified (not in Page 10).

*We thank the reviewer very much for this comment. Since the STR expansion was a special type of inversion, we annotated all the detected insertions using TRF (4.09) and RepeatMasker to see whether they were STR or other known repeat classes. We have added the text “Insertions were further annotated as tandem repeats or known repeat classes using TRF (4.09) and RepeatMasker (4.1).” into the “Alignment and SVs calling” section on Page 5.*

5, While it is interesting, I do not feel that the entire section of “Measurement of telomere repeat length” is rigorously performed. Alignment to reference genome is unlikely to characterize telomere length accurately or comprehensively, and a simple counting of TTAGGG motif is also not sufficient to characterize telomere. Additionally, this is an area that can greatly benefit from improvements in basecalling (as I mentioned above), yet they used an older version of basecaller. For these reasons, I have confidence on the results and I feel it is best to remove this entire part from the paper so it focuses on SV detection. Certainly the authors can feel free to perform analysis on telomere length, but more sophisticated methods (than simple mapping to reference genome) are definitely needed to obtain reliable results.

*We thank the reviewer very much for this suggestion. We have removed this part from the revised manuscript.*

6, I think the novel gene fusions are the highlights of the manuscript. The authors have done a good job of validating its existence and characterizing its functional effects. One natural question arising from the results is that whether such gene fusions are recurrent (possibly at low frequency) in the population. There are many public databases on short-read RNA-seq that they may be able to check and see if similar ones has been observed (but not confirmed, due to low expression levels), and I assume that the authors may have access to additional CRC samples and can perform a population-wide screen using PCR to see whether the fusions can be observed in other samples.

*We thank the reviewer very much for the comments. We have checked several fusion gene databases (including FusionGDB, TICdb, tumorfusions.org and COSMIC), but the gene fusions (RNF38-RAD51B and SMAD3-SHISA6) were not in any of these databases. The RNF38-RAD51B gene fusion was also not detected in 103 CRC tumor samples via PCR method. Thus, the frequencies of RNF38-RAD51B and SMAD3-SHISA6 gene fusions may be very low in population.*

7, I have similar concerns on the methylation analysis in this manuscript. It is somewhat superficial, and there is no paired methylation microarray data to support the computational analysis. Hypomethylation was observed, but it is as expected and is well known, and the level cited in the paper (“12 out of 21 CRC samples with the average 63%”) casts doubt on the reliability of the methylation caller. As the paper focused on SVs, I feel this part does not need to be included in the paper unless a substantially different and more rigorous analysis is done with novel biological insights.

*We thank the reviewer very much for this suggestion. We have removed this part from the revised manuscript.*

8, RNA-Seq and functional assays (including animal studies) are important for this type of studies to assay SVs in a genome-wide scale. I am glad that the authors have performed these additional assays to support findings on long-read sequencing, but I feel they should include some descriptions in the Abstract so that the paper feels more comprehensive than merely an explorative sequencing study.

*We thank the reviewer very much for the comment. We have added the texts “…confirmed by RNA-seq…” and “confirmed by in vitro migration and invasion assay, and in vivo metastasis experiments.” to the Abstract.*

**Have all data underlying the figures and results presented in the manuscript been provided?**
Large-scale datasets should be made available via a public repository as described in the *PLOS Genetics* [data availability policy](http://journals.plos.org/plosgenetics/s/data-availability), and numerical data that underlies graphs or summary statistics should be provided in spreadsheet form as supporting information.

Reviewer #1: **No:** Missing information that would be needed to support results. E.g. information describing the short-read DNA and RNA seq, methylation analysis, in-vitro experiments.

Reviewer #2: None

*We thank the reviewers very much for the comments. We have added the numerical data that underlies graphs or summary statistics into new Table S4.*
