# Supplementary material for: Long-read sequencing identifies novel structural variations in colorectal cancer
Source: PLoS Genet. 2023 Feb 22;19(2):e1010514. doi: 10.1371/journal.pgen.1010514 (PMC10013895; doi:10.1371/journal.pgen.1010514)
Supplement: Attachment — Submitted filename: Response.docx [file pgen.1010514.s018.docx]

*Union Hospital, Tongji Medical College, Huazhong University of Science and Technology,*

*1277 Jiefang Avenue, Wuhan, China 430022*

Dear *Editor*,

We thank you very much for your and reviewer’s constructive comments and suggestions on our manuscript entitled “*Long-read sequencing identifies novel structural variations in colorectal cancer*”. We have carefully studied the comments and suggestions, and have revised the manuscript accordingly. Revised portions are highlighted in red in the manuscript. We would like to express our sincere appreciation to you and reviewers for comments and suggestions on our manuscript.

Your sincerely,

**Zheng Wang, MD/ PhD**

*Professor of Medicine,*

*Department of Gastrointestinal Surgery,*

*Research Center for Tissue Engineering and Regenerative Medicine,*

*Union Hospital, Tongji Medical College,*

*Huazhong University of Science and Technology*

*1277 Jiefang Ave., Wuhan, China 430022*

*zhengwang@hust.edu.cn*

*Tel: +86-27-85726612*

**Lin Wang, PhD**

*Professor and Director,*

*Research Center for Tissue Engineering and Regenerative Medicine,*

*Department of Clinical Laboratory,*

*Union Hospital, Tongji Medical College,*

*Huazhong University of Science and Technology*

*1277 Jiefang Ave., Wuhan, China 430022*

*lin_wang@hust.edu.cn*

*Tel: +86-27-85726612*

**Journal**: PLOS Genetics

**Manuscript ID**: PGENETICS-D-21-01626

**Title**: Long-read sequencing identifies novel structural variations in colorectal cancer

**Responses to Reviewers’ Comments**

*Thank the reviewers very much for the comments and suggestions that greatly help make this manuscript much better!*

**Reviewer #2:** The authors have addressed all my comments.

I find the improvement of accuracy described in the statement "Compared with Guppy 2.0.3 (88.2%), the average mapping identity of Guppy 6.0.1 analyzed data improved to be 97.7%" quite impressive. The authors should provide details how the measure the "average mapping identity" (for example, did they look into MD tag or CIGR string of BAM file or use a publicly available tool to calculate mapping accuracy?)

*We thank the reviewer very much for the comments. We used minimap2 for the alignment of reads. The minimap2 outputs have a new tag de:f, which gives an estimate of sequence divergence. The mapping identity of Guppy 6.0.1 super accuracy model analyzed data was calculated as "1-divergence" using tag de:f (https://github.com/lh3/minimap2/issues/281).*

The same issue is in Figure 1: the mapping identity looks very low (all samples seem to have <88% identity, and some of them even have <85% identity). This may be due to how the identity is actually calculated, and how are the indels handled in the calculation. Therefore, it is important for the authors to provide more details on the calculation.

*There was indeed a difference in the identity calculation method. In our article, the results of samtools stats will output the statistics of the error rate (mismatches / bases mapped), and we directly used 1-error rate to represent mapping identity.*

*However, compared with the old versions, the accuracy of Guppy 6.0.1 super accuracy model analyzed data is significantly improved. For instance, D Beyter et al. sequenced 3622 Icelanders, and it can be seen from their Supplementary Figure 1 (see below， Figure R1) that most of the alignment error rates analyzed by the guppy V3 hac model were higher than 10% (Nat Genet 53, 779–786 (2021). Doi: 10.1038/s41588-021-00865-4).*

***Figure R1.*** *Screenshot from Nat Genet 53, 779–786 (2021).*

*In contrast, EPI2ME labs released GM24385 Datasets which were rebasecalled with Guppy 5.0.6 super accuracy model (https://labs.epi2me.io/gm24385_2021.05/). It can be seen from the figure below (Figure R2) that the mode of alignment accuracy is about 97%.*

***Figure R2.*** *Screenshot from EPI2ME labs (https://labs.epi2me.io/gm24385_2021.05/)*

Figure 3: "Sequence analysis of somatic deletions/ insertions and methylation levels of mobile elements". Since methylation analysis is not included in the manuscript I think this is wrong.

*We thank the reviewer very much for pointing this mistake out. We have changed the legend of Figure 3 to “Sequence analysis of somatic deletions/ insertions.”.*

Also there is a Table S4 (raw data for figures) but I do not see any other identifiers of data sets. There was an editorial question "have all data underlying the figures and results presented in the manuscript been provided" and I am not sure if this refers to FASTQ files, FAST5 files, or a set of summary files for Figures. In any case, since these are tumor samples the authors can consider sharing the raw data from sequencing (FAST5 files) depending on journal and funding agency requirement.

*We thank the reviewer very much for the comments. Since the volume of FAST5 files were too large (>40 TB) to share through databases, we uploaded the raw FASTQ files to the Genome Sequence Archive (GSA) in the China National Center for Bioinformation (CNCB, https://ngdc.cncb.ac.cn/gsa-human/browse/HRA002638).*

There is no mention of "public access to data" in the manuscript. This is an editorial question that should be addressed: how public can access the data.

*The sequence data were deposited in the Genome Sequence Archive (GSA) in the China National Center for Bioinformation (CNCB), under accession number HRA002638, that are publicly accessible (https://ngdc.cncb.ac.cn/gsa-human/browse/HRA002638).*

**Reviewer #3:**

General

In this manuscript, the authors describe the whole genome sequencing analysis of 21 colorectal cancers (WGS) using PromethION. The sequencing was performed in TN pairs and somatic structural variation (SVs) were identified by their subtractions. An average of 494 SVs per sample or a total of 5,200 novel somatic SVs were identified. The identified SVs included those which altered the gene structure or gene expressions of key suppressor genes such as APC and CFTR. In addition, they identified a novel fusion gene, RNF38-RAD51B, which may be the highlight of this paper. They attempted demonstrate that this gene fusion enhances favorable features of cancer cells, such as cellular migration, invasion, and metastasis abilities. To demonstrate these functions, the authors employed a cell culture and a mouse metastasis model. Overall, I have no doubt that the Nanopore sequencing can be used for cancer genome analysis and will shed a new light on the cancer mutation from a yet different angle than the current short read sequencing. In fact, several previous papers have already demonstrated its power. With such a circumstance, the collected datasets should be valuable for further future studies as a resource. On the other hand, I have to point out that the biological findings or genomic characterization described here is not sufficiently relevant. Therefore, I recommend this paper should shift the focus more on the value of the generated dataset itself or biological findings deduced from the analysis of the collected data.

Major comments:

1. Even though I admit the primary value of this paper lies in its dataset itself, at least, some basic characterization should be conducted. For example, the authors should scrutinize the abundance and the patterns of the SVs between for each sub-category, such as MSI-H and MSS types, different stages and so on, in more details. Even though the sample number may not be sufficient, so that the difference may not be always statistically significant, the results could be shown for individual cases with given pathological subtypes of the specimens. The results shown in Figure 2C is too superficial.

*We thank the reviewer very much for the comments. We compared the number of each type of SVs (excluding insertions in STR) between different MSI status and different stages, respectively. The results showed that the number of inversions in MSI-H samples was significantly lower than that in MSS samples (p=0.0476). The numbers of other types of SVs were similar between different MSI status and different stages. We have added the result as new figure S5, and added the text “The number of inversions in MSI-H samples was significantly lower than that in MSS samples, and the numbers of other types of SVs were across between different MSI status and different stages” at the “Long-read sequencing identified widespread somatic SVs in colorectal cancer” section on Page 9.*

2. Comparison with the short read sequence data should be further enriched. Especially, I’m curious about the proportion of the inconsistent detections depending on the variant allele frequencies (VAFs). For the purpose of comparison, WES may not be appropriate, except for earning the sequencing depth, thus, the short read WGS should be conducted for some cases,

*We thank the reviewer very much for the advice. Many published papers have reported the advantages of long-read sequencing over short-read sequencing in detecting germline and somatic structural variations. Therefore, in this study, we did not design a comparison between the short-read WGS and the long-read WGS. Further comparisons will be made when we sequence the WGS data in next study, especially regarding the proportion of the inconsistent detections depending on the variant allele frequencies (VAFs).*

Minor comments:

3. There is no substantial novelty about the method. All the procedures are the standard ones; NGMLR is used for mapping, Sniffles (version 1.0.8) is used for SV detection, and normal detection results are subtracted from tumor detection results; As a threshold, the average depth of support reads on the tumor side is 0.3 or more (approximately five or more), and on the normal side, two or more support reads are required. All of these procedures are, more or less, the previously scrutinized ones, thus, has been technically validated, except for the following points.

i. While the minimum SV size is 50 bp, the maximum margin for merging SVs is set to 1,000 bp, which is arbitrary to this study. Particularly, I wonder whether it causes some errors where some of the multiple SVs may be merged into one SV or one SVs are divided into multiple SVs. Therefore, the size and mutual distance of the detected SVs should be presented somewhere.

*We thank the reviewer very much for the comments.* *We calculated the distribution of SV lengths and the mutual distances of the detected SVs. The results showed that the lengths of most SVs (>98%) were less than 10,000 bp, and the mutual distance of most SVs were between 10^5^ ~ 10^7^ bp. Thus, we believed that only very few of SVs would be merged into one SV or divided into multiple SVs. We have added the results as new Figure S2, and added the text “The lengths of 98% of SVs were less than 10,000 bp, and the mutual distance of most SVs (~80%) was between 10^5^ ~ 10^7^ bp” at the “Long-read sequencing identified widespread somatic SVs in colorectal cancer” section on Page 8.*

ii. Unless there is a particular reason for using hg37, instead of hg38, the reference should be updated. It is also preferable if the authors can also consider using the recent more completed genome (“T2T genome”; Science 2022) as a reference to see the difference in the results.

*We thank the reviewer very much for the advice. In this study, we attempted to use our local germline SV database (unpublished, containing the nanopore WGS data of ~500 healthy donors) to eliminate false-positive somatic SVs. Since the germline SVs were generated using hg37 as a reference genome, we decided to use hg37 in this study. To discovery more novel cancer-related SVs, we will use the updated reference genomes (such as T2T genome) in further studies.*

iii. So is the version of Sniffles. The developer mentions that Sniffles2 is better (https://www.biorxiv.org/content/10.1101/2022.04.04.487055v1.full.pdf)

*We thank the reviewer very much for the advice. Sniffles2 enables the detection of low-frequency SVs and population-scale SVs. We tested the performance of Sniffles2 in a pair of samples, and the results showed that the Sniffles2 detected more SVs than the old version. In this study, we have manually checked 14,508 candidate somatic SVs to ensure the accuracy of SVs and finally 5200 SVs were finally identified. We will use new tools to analyze our data in further studies, and add more somatic SVs to our databases.*

4. Evidence for the biological or clinical relevance of the detected fusion-gene SV (RNF38-RAD51B) is not sufficiently solid as follows, Therefore, the related descriptions should be toned down.

*We thank the reviewer very much for the comment. We have revised the description of this gene fusion in the discussion.*

i. Supporting evidence shown in this paper for the driver function of the new fusion gene is only from several cell lines and a relatively artificial mouse model. Therefore, further supportive literal evidences should be enriched in the discussion section. I’m also curious about the VAF of this fusion in this specimen, so that its driver role is ensured.

*We thank the reviewer very much for the comments. We have added the text “our work uncovered a novel gene fusion, RNF38-RAD51B, which could enhance CRC cells’ oncogenic functions. RNF38 was reported as a vital driver of cancer progression and could promote the invasion and metastasis of cancer cells (30,31). The RNF38-RAD51B gene fusion may enhance the expression or function of RNF38, since it significantly promoted the invasion and metastasis ability of colorectal cancer cells. Although the molecular mechanisms and clinical relevance of this gene fusion need to be further studied, our results suggest that nanopore sequencing may serve as a new strategy for detecting oncogenic gene fusions” at the “Discussion” section on Page 12.*

ii. Please estimate the sufficient expression level of the RNF38-RAD51B fusion gene. To my knowledge, usually the fusion gene transcripts, such as those of the ALK, RET or ROS-1 fusion genes, show substantial expression levels as of the RNA seq analysis and those expression levels can be estimated, once the precise junction is identified. It is preferable in the downstream gene expression levels can be also analyzed further. This information is important to ensure that this gene should truly realize its driver role at the given transcript level.

*We thank the reviewer very much for the comments. Besides nanopore sequencing, the RNF38-RAD51B gene fusion was also detected by RNA sequencing, and the result showed that the junction reads count was 52 and the spanning fragments count was 2.*

*The upregulated RNF38 expression was found to promote cancer cell invasion and metastasis by inducing cell epithelial-mesenchymal transition (EMT) (J Cancer. 2018;9:841–50; J Exp Clin Cancer Res. 2019; 1:113). We examined the RNA expression levels of EMT markers SNA1 and CDH1. The results showed that the expression of SNA1 was significantly increased in tumor sample (cancer: 2.708; normal: 0.426, FPKM), and the expression of CDH1 was reduced (cancer: 85.302; normal: 101.597, FPKM), consistent with previously reported (J Exp Clin Cancer Res. 2019; 1:113).*

iii. I wonder how frequent this fusion gene occurs among the patients of CRCs. Also, please conduct a short read sequencing analysis to see whether this fusion could not be found by the current short read approach even at a sufficient sequencing depth, possibly by WGS.

*We thank the reviewer very much for the comments. The RNF38-RAD51B fusion gene was also detected by short-read transcriptome sequencing. We have checked several fusion gene databases (including FusionGDB, TICdb, tumorfusions.org and COSMIC); the RNF38-RAD51B gene fusion was not in any of these databases. We also tried PCR method to detect this gene fusion, but it was not detected in 103 CRC tumor samples. Thus, the frequencies of RNF38-RAD51B and SMAD3-SHISA6 gene fusions may be very low in population.*

5. Figure 2D: I’m not sure what the “recurrence” (on X-axis) indicates. Please specify the definition (SVs within 1,000 bp is regarded as “identical?”) and its relevance. For example, even though most of these SVs should not have any biological role, is it possible that they may represent hot spots? Or are they merely following the decreasing curve as theoretically supposed?

*We thank the reviewer very much for the comments. The “recurrence” refers to the number of samples in which an SV is detected. We have added the text “The “Recurrence” on X-axis refers to the number of samples in which an SV is detected” at the figure legend of Figure 2D.*

*To identify recurrent SVs, we regarded large SVs (> 10 kbp) within 1,000 bp as identical. For small SVs (< 1,000 bp), the identical somatic SVs were identified by manually checking the alignment files of each SV (described in “materials and methods” section). Thus, the recurrence number of somatic SVs was reliable, and the SVs with high recurrence numbers could be regarded as hot spots.*

Miscellaneous comments:

6. In the reply to another reviewer, the authors stated that further verification of methylation detection from the long reads was necessary, so the related contents were deleted from this paper. However, several papers have already assured the detection power or the limit of the this approach (see https://genomebiology.biomedcentral.com/articles/10.1186/s13059-021-02510-z). Was the sequencing depth of other factor insufficient to be used for the methylation call? The reason of the withdraw should be further specified.

*We thank the reviewer very much for the comments. The main reason we removed the methylation analysis was that the findings from that analysis were expected and known. Besides, the lack of bisulphite sequencing verification also made the conclusions were not quite reliable.*

7. In the Introduction section (line 15, page 3), “single-molecule, read-time sequencing (SMAT)” should be “single-molecule real-time sequencing (SMRT)”?

*We thank the reviewer very much for pointing this mistake out. We have changed the text “single-molecule, read-time sequencing (SMAT)” to “single-molecule read-time sequencing (SMRT)” on Page 3.*

8. Perhaps the most important issue is the data availability. Please make sure all the raw data will be available via the controlled access. I wonder if the data deposition to CNCB is admitted according to the journal policy.

*We thank the reviewer very much for the comments, The sequence data were deposited in the Genome Sequence Archive (GSA) in the China National Center for Bioinformation (CNCB), under accession number HRA002638, that are publicly accessible (*[*https://ngdc.cncb.ac.cn/gsa-human/browse/HRA002638*](https://ngdc.cncb.ac.cn/gsa-human/browse/HRA002638)*). We will make sure the data are accessible for all researchers.*
