# Supplementary material for: Long-read sequencing identifies novel structural variations in colorectal cancer
Source: PLoS Genet. 2023 Feb 22;19(2):e1010514. doi: 10.1371/journal.pgen.1010514 (PMC10013895; doi:10.1371/journal.pgen.1010514)
Supplement: Peer review history

# Peer review: Decision Letter 0

**DOI:** 10.1371/journal.pgen.1010514.r001

**Authors:** David J. Kwiatkowski, Richarda de Voer

7 Mar 2022

Dear Dr Wang,

Thank you very much for submitting your Research Article entitled 'Long-read sequencing identifies novel structural variations in colorectal cancer' to PLOS Genetics.

The manuscript was fully evaluated at the editorial level and by independent peer reviewers. The reviewers appreciated the attention to an important problem, but raised some substantial concerns about the current manuscript. In many instances, there is a lack of important detail.  In particular, the comparison between the short-read DNA sequencing and the RNAseq analysis requires additional information. Furthermore, as highlighted by both reviewers below it would be of interest to include genome-wide methylation analysis in the manuscript. Based on the reviews, we will not be able to accept this version of the manuscript, but we would be willing to review a much-revised version. We cannot, of course, promise publication at that time.

Should you decide to revise the manuscript for further consideration here, your revisions should address the specific points made by each reviewer. We will also require a detailed list of your responses to the review comments and a description of the changes you have made in the manuscript.

If you decide to revise the manuscript for further consideration at PLOS Genetics, please aim to resubmit within the next 60 days, unless it will take extra time to address the concerns of the reviewers, in which case we would appreciate an expected resubmission date by email to plosgenetics@plos.org.

If present, accompanying reviewer attachments are included with this email; please notify the journal office if any appear to be missing. They will also be available for download from the link below. You can use this link to log into the system when you are ready to submit a revised version, having first consulted our Submission Checklist.

To enhance the reproducibility of your results, we recommend that you deposit your laboratory protocols in protocols.io, where a protocol can be assigned its own identifier (DOI) such that it can be cited independently in the future. Additionally, PLOS ONE offers an option to publish peer-reviewed clinical study protocols. Read more information on sharing protocols at https://plos.org/protocols?utm_medium=editorial-email&utm_source=authorletters&utm_campaign=protocols

Please be aware that our data availability policy requires that all numerical data underlying graphs or summary statistics are included with the submission, and you will need to provide this upon resubmission if not already present. In addition, we do not permit the inclusion of phrases such as "data not shown" or "unpublished results" in manuscripts. All points should be backed up by data provided with the submission.

While revising your submission, please upload your figure files to the Preflight Analysis and Conversion Engine (PACE) digital diagnostic tool.  PACE helps ensure that figures meet PLOS requirements. To use PACE, you must first register as a user. Then, login and navigate to the UPLOAD tab, where you will find detailed instructions on how to use the tool. If you encounter any issues or have any questions when using PACE, please email us at figures@plos.org.

PLOS has incorporated Similarity Check, powered by iThenticate, into its journal-wide submission system in order to screen submitted content for originality before publication. Each PLOS journal undertakes screening on a proportion of submitted articles. You will be contacted if needed following the screening process.

To resubmit, use the link below and 'Revise Submission' in the 'Submissions Needing Revision' folder.

[LINK]

We are sorry that we cannot be more positive about your manuscript at this stage. Please do not hesitate to contact us if you have any concerns or questions.

Yours sincerely,

Richarda de Voer

Guest Editor

PLOS Genetics

David Kwiatkowski

Section Editor: Cancer Genetics

PLOS Genetics

Reviewer's Responses to Questions

**Comments to the Authors:**

**Please note here if the review is uploaded as an attachment.**

Reviewer #1: The 'Long-read sequencing identifies novel structural variations in colorectal cancer' describes the assessment of long read sequencing (ONT) as a method to investigate somatic SVs in 21 colorectal cancer samples. The data presented has useful insights into the use of ONT for the detection of somatic SVs. However, many of the methods and results lack sufficient details and make it difficult to interpret the findings. While I believe the data generated for the manuscript has merit and would be of interest to the community, I think a significant amount of additional information describing the data and revisions to the manuscript to improve its clarity are required before it is suitable for publication.

I would highly recommend English editing for the manuscript to improve grammar and clarity. While the manuscript is readable there are sentences and paragraphs that are especially unclear that make interpretation of results difficult.

Introduction

• I think the claim that short read sequencing has high false positive rates should be softened or justified as some callers have been shown to have high precision.

• The final paragraph highlighting the findings is particularly hard to follow.

Methods

• It is unclear how the manual curation of SVs was conducted and what was specifically was being curated.

• It is unclear if and why different reference genomes were used.

• How variants were annotated is unclear. Databases should include version numbers.

• The information specific to the in vitro work is severely lacking. I would have expected to see basic information describing cell culture and experimental conditions would be included (E.g. Where cells came from, growth conditions, media…) as well as more experimental information (E.g. how many replicates per experiment). More details about the mice and the tumours would also benefit the manuscript.

Results

• It is unclear if all samples were sequenced and analysed with each different technology (Short read DNA and RNA and ONT?). This should also be addressed in the methods.

• Details describing the short DNA or RNA sequencing need to be included. There is no mention of sequencing depth or quality for either and no comparison between is provided. E.g. if SVs were also called by short read sequencing, what is the overlap?

• Addition of genome wide methylation results/findings would benefit the manuscript.

• I would have liked to see a breakdown of variants selected for and filtered out for a paper that is evaluating ONT SV calling. E.g. how many variants were filtered based on the different criteria.

• The SVs are described as high confidence however no evidence or justification of this given.

• If the over expressing cell lines were created, details about this should be included and characteristics of these cells should be described (E.g. what vector, what level of overexpression). This also applies to methods.

• I think the finding of the fusion event and methylation patterns is interesting, but it should be highlighted as an example finding, given that no other events were reported for the majority of the 21 cases.

Discussion

• The discussion is reasonably fair but would benefit from English editing.

Other comments

1. Primer sequences should be included and I would have liked to see Sanger chromatograms in supplementary to show breakpoints of the fusion.

Reviewer #2: This is a study that uses long-read sequencing to identify structural variants from colorectal cancer (CRC). The study used 21 pairs of CRC samples on Nanopore whole-genome long-read sequencing which is an impressive sample size for this type of studies. In addition to novel structural variants, they also identified two novel gene fusions that may be of clinical significance. Overall this is a unique study with relatively large sample size and appropriate functional validation, further demonstrating the power of long-read sequencing in the understanding of the human genome. My comments are below to help improve the manuscript:

1, The introduction section needs expansion to give appropriate background to readers who are not familiar with long-read sequencing. In particular, several different techniques for long-read sequencing need to be summarized, before focusing on Nanopore sequencing. Additionally, quite a number of manuscripts are published over the past few years illustrating the power of long-read (some used Nanopore) sequencing to identify likely pathogenic SVs that are missed by short-read sequencing, and I think these studies should be mentioned, even though the prior studies focused on genetic diagnosis of Mendelian diseases rather than somatic driver mutations in cancer. The relative merits, including cost, speed, DNA requirements, etc should be slightly described or discussed as well. These will give readers who are not familiar with the field the necessary background information to appreciate the study.

2, it may have been mentioned elsewhere in the paper, but I cannot find a detailed description of the “21 pairs of tumor samples and matched para-carcinoma samples”. How are they ascertained, how are the samples retrieved, and are they freshly frozen? What are the pathological findings on these patients? These are important questions that need to be addressed.

3, Since Guppy 6 is already available today, I would suggest the authors to at least basecall a couple of samples to see whether it makes a difference in terms of detecting the SVs highlighted in the paper. My guess is that the difference on SVs may be minor since the different Guppy improved basecalling accuracy for SNP/indel calling, but it would be good to see this more quantitatively.

4, The study included five pairs of MSI-H samples, which as expected has abnormal expansion of short tandem repeat (STR) regions (Figure 2C). This is an interesting aspect that there is clear advantage of long-read sequencing over short-read sequencing, but I do not see much technical details on how the STR expansion is identified and quantified (not in Page 10).

5, While it is interesting, I do not feel that the entire section of “Measurement of telomere repeat length” is rigorously performed. Alignment to reference genome is unlikely to characterize telomere length accurately or comprehensively, and a simple counting of TTAGGG motif is also not sufficient to characterize telomere. Additionally, this is an area that can greatly benefit from improvements in basecalling (as I mentioned above), yet they used an older version of basecaller. For these reasons, I have confidence on the results and I feel it is best to remove this entire part from the paper so it focuses on SV detection. Certainly the authors can feel free to perform analysis on telomere length, but more sophisticated methods (than simple mapping to reference genome) are definitely needed to obtain reliable results.

6, I think the novel gene fusions are the highlights of the manuscript. The authors have done a good job of validating its existence and characterizing its functional effects. One natural question arising from the results is that whether such gene fusions are recurrent (possibly at low frequency) in the population. There are many public databases on short-read RNA-seq that they may be able to check and see if similar ones has been observed (but not confirmed, due to low expression levels), and I assume that the authors may have access to additional CRC samples and can perform a population-wide screen using PCR to see whether the fusions can be observed in other samples.

7, I have similar concerns on the methylation analysis in this manuscript. It is somewhat superficial, and there is no paired methylation microarray data to support the computational analysis. Hypomethylation was observed, but it is as expected and is well known, and the level cited in the paper (“ 12 out of 21 CRC samples with the average 63%”) casts doubt on the reliability of the methylation caller. As the paper focused on SVs, I feel this part does not need to be included in the paper unless a substantially different and more rigorous analysis is done with novel biological insights.

8, RNA-Seq and functional assays (including animal studies) are important for this type of studies to assay SVs in a genome-wide scale. I am glad that the authors have performed these additional assays to support findings on long-read sequencing, but I feel they should include some descriptions in the Abstract so that the paper feels more comprehensive than merely an explorative sequencing study.

**********

**Have all data underlying the figures and results presented in the manuscript been provided?**

Large-scale datasets should be made available via a public repository as described in the *PLOS Genetics* data availability policy, and numerical data that underlies graphs or summary statistics should be provided in spreadsheet form as supporting information.

Reviewer #1: **No: **Missing information that would be needed to support results. E.g. information describing the short-read DNA and RNA seq, methylation analysis, in-vitro experiements.

Reviewer #2: None

**********

PLOS authors have the option to publish the peer review history of their article (what does this mean?). If published, this will include your full peer review and any attached files.

If you choose “no”, your identity will remain anonymous but your review may still be made public.

**Do you want your identity to be public for this peer review?** For information about this choice, including consent withdrawal, please see our Privacy Policy.

Reviewer #1: No

Reviewer #2: No

# Author comment: Author response to Decision Letter 0

**DOI:** 10.1371/journal.pgen.1010514.r002

14 Jul 2022

Attachment Submitted filename: Response to the reviewers.docx Click here for additional data file.

# Peer review: Decision Letter 1

**DOI:** 10.1371/journal.pgen.1010514.r003

**Authors:** David J. Kwiatkowski, Richarda de Voer

15 Aug 2022

Dear Dr Wang,

Thank you very much for submitting your Research Article entitled 'Long-read sequencing identifies novel structural variations in colorectal cancer' to PLOS Genetics.

The manuscript was fully evaluated at the editorial level and by independent peer reviewers. The reviewers appreciated the attention to an important topic but identified some concerns that we ask you address in a revised manuscript

We therefore ask you to modify the manuscript according to the review recommendations. Your revisions should address the specific points made by each reviewer.

In addition we ask that you:

1) Provide a detailed list of your responses to the review comments and a description of the changes you have made in the manuscript.

2) Upload a Striking Image with a corresponding caption to accompany your manuscript if one is available (either a new image or an existing one from within your manuscript). If this image is judged to be suitable, it may be featured on our website. Images should ideally be high resolution, eye-catching, single panel square images. For examples, please browse our archive. If your image is from someone other than yourself, please ensure that the artist has read and agreed to the terms and conditions of the Creative Commons Attribution License. Note: we cannot publish copyrighted images.

We hope to receive your revised manuscript within the next 30 days. If you anticipate any delay in its return, we would ask you to let us know the expected resubmission date by email to plosgenetics@plos.org.

If present, accompanying reviewer attachments should be included with this email; please notify the journal office if any appear to be missing. They will also be available for download from the link below. You can use this link to log into the system when you are ready to submit a revised version, having first consulted our Submission Checklist.

While revising your submission, please upload your figure files to the Preflight Analysis and Conversion Engine (PACE) digital diagnostic tool. PACE helps ensure that figures meet PLOS requirements. To use PACE, you must first register as a user. Then, login and navigate to the UPLOAD tab, where you will find detailed instructions on how to use the tool. If you encounter any issues or have any questions when using PACE, please email us at figures@plos.org.

Please be aware that our data availability policy requires that all numerical data underlying graphs or summary statistics are included with the submission, and you will need to provide this upon resubmission if not already present. In addition, we do not permit the inclusion of phrases such as "data not shown" or "unpublished results" in manuscripts. All points should be backed up by data provided with the submission.

To enhance the reproducibility of your results, we recommend that you deposit your laboratory protocols in protocols.io, where a protocol can be assigned its own identifier (DOI) such that it can be cited independently in the future. Additionally, PLOS ONE offers an option to publish peer-reviewed clinical study protocols. Read more information on sharing protocols at https://plos.org/protocols?utm_medium=editorial-email&utm_source=authorletters&utm_campaign=protocols

Please review your reference list to ensure that it is complete and correct. If you have cited papers that have been retracted, please include the rationale for doing so in the manuscript text, or remove these references and replace them with relevant current references. Any changes to the reference list should be mentioned in the rebuttal letter that accompanies your revised manuscript. If you need to cite a retracted article, indicate the article’s retracted status in the References list and also include a citation and full reference for the retraction notice.

PLOS has incorporated Similarity Check, powered by iThenticate, into its journal-wide submission system in order to screen submitted content for originality before publication. Each PLOS journal undertakes screening on a proportion of submitted articles. You will be contacted if needed following the screening process.

To resubmit, you will need to go to the link below and 'Revise Submission' in the 'Submissions Needing Revision' folder.

[LINK]

Please let us know if you have any questions while making these revisions.

Yours sincerely,

Richarda de Voer

Guest Editor

PLOS Genetics

David Kwiatkowski

Section Editor

PLOS Genetics

Reviewer's Responses to Questions

**Comments to the Authors:**

**Please note here if the review is uploaded as an attachment.**

Reviewer #2: The authors have addressed all my comments.

I find the improvement of accuracy described in the statement "Compared with Guppy 2.0.3 (88.2%), the average mapping identity of Guppy 6.0.1 analyzed data improved to be 97.7%" quite impressive. The authors should provide details how the measure the "average mapping identity" (for example, did they look into MD tag or CIGR string of BAM file or use a publicly available tool to calculate mapping accuracy?)

The same issue is in Figure 1: the mapping identity looks very low (all samples seem to have <88% identity, and some of them even have <85% identity). This may be due to how the identity is actually calculated, and how are the indels handled in the calculation. Therefore, it is important for the authors to provide more details on the calculation.

Figure 3: "Sequence analysis of somatic deletions/ insertions and methylation levels of mobile elements". Since methylation analysis is not included in the manuscript I think this is wrong.

Also there is a Table S4 (raw data for figures) but I do not see any other identifiers of data sets. There was an editorial question "have all data underlying the figures and results presented in the manuscript been provided" and I am not sure if this refers to FASTQ files, FAST5 files, or a set of summary files for Figures. In any case, since these are tumor samples the authors can consider sharing the raw data from sequencing (FAST5 files) depending on journal and funding agency requirement.

There is no mention of "public access to data" in the manuscript. This is an editorial question that should be addressed: how public can access the data.

Reviewer #3: General

In this manuscript, the authors describe the whole genome sequencing analysis of 21 colorectal cancers (WGS) using PromethION. The sequencing was performed in TN pairs and somatic structural variation (SVs) were identified by their subtractions. An average of 494 SVs per sample or a total of 5,200 novel somatic SVs were identified. The identified SVs included those which altered the gene structure or gene expressions of key suppressor genes such as APC and CFTR. In addition, they identified a novel fusion gene, RNF38-RAD51B, which may be the highlight of this paper. They attempted demonstrate that this gene fusion enhances favorable features of cancer cells, such as cellular migration, invasion, and metastasis abilities. To demonstrate these functions, the authors employed a cell culture and a mouse metastasis model. Overall, I have no doubt that the Nanopore sequencing can be used for cancer genome analysis and will shed a new light on the cancer mutation from a yet different angle than the current short read sequencing. In fact, several previous papers have already demonstrated its power. With such a circumstance, the collected datasets should be valuable for further future studies as a resource. On the other hand, I have to point out that the biological findings or genomic characterization described here is not sufficiently relevant. Therefore, I recommend this paper should shift the focus more on the value of the generated dataset itself or biological findings deduced from the analysis of the collected data.

Major comments:

1. Even though I admit the primary value of this paper lies in its dataset itself, at least, some basic characterization should be conducted. For example, the authors should scrutinize the abundance and the patterns of the SVs between for each sub-category, such as MSI-H and MSS types, different stages and so on, in more details. Even though the sample number may not be sufficient, so that the difference may not be always statistically significant, the results could be shown for individual cases with given pathological subtypes of the specimens. The results shown in Figure 2C is too superficial.

2. Comparison with the short read sequence data should be further enriched. Especially, I’m curious about the proportion of the inconsistent detections depending on the variant allele frequencies (VAFs). For the purpose of comparison, WES may not be appropriate, except for earning the sequencing depth, thus, the short read WGS should be conducted for some cases,

Minor comments:

3. There is no substantial novelty about the method. All the procedures are the standard ones; NGMLR is used for mapping, Sniffles (version 1.0.8) is used for SV detection, and normal detection results are subtracted from tumor detection results; As a threshold, the average depth of support reads on the tumor side is 0.3 or more (approximately five or more), and on the normal side, two or more support reads are required. All of these procedures are, more or less, the previously scrutinized ones, thus, has been technically validated, except for the following points.

i. While the minimum SV size is 50 bp, the maximum margin for merging SVs is set to 1,000 bp, which is arbitrary to this study. Particularly, I wonder whether it causes some errors where some of the multiple SVs may be merged into one SV or one SVs are divided into multiple SVs. Therefore, the size and mutual distance of the detected SVs should be presented somewhere.

ii. Unless there is a particular reason for using hg37, instead of hg38, the reference should be updated. It is also preferable if the authors can also consider using the recent more completed genome (“T2T genome”; Science 2022) as a reference to see the difference in the results.

iii. So is the version of Sniffles. The developer mentions that Sniffles2 is better (https://www.biorxiv.org/content/10.1101/2022.04.04.487055v1.full.pdf)

4. Evidence for the biological or clinical relevance of the detected fusion-gene SV (RNF38-RAD51B) is not sufficiently solid as follows, Therefore, the related descriptions should be toned down.

i. Supporting evidence shown in this paper for the driver function of the new fusion gene is only from several cell lines and a relatively artificial mouse model. Therefore, further supportive literal evidences should be enriched in the discussion section. I’m also curious about the VAF of this fusion in this specimen, so that its driver role is ensured.

ii. Please estimate the sufficient expression level of the RNF38-RAD51B fusion gene. To my knowledge, usually the fusion gene transcripts, such as those of the ALK, RET or ROS-1 fusion genes, show substantial expression levels as of the RNA seq analysis and those expression levels can be estimated, once the precise junction is identified. It is preferable in the downstream gene expression levels can be also analyzed further.This information is important to ensure that this gene should truly realize its driver role at the given transcript level.

iii. I wonder how frequent this fusion gene occurs among the patients of CRCs. Also, please conduct a short read sequencing analysis to see whether this fusion could not be found by the current short read approach even at a sufficient sequencing depth, possibly by WGS.

5. Figure 2D: I’m not sure what the “recurrence” (on X-axis) indicates. Please specify the definition (SVs within 1,000 bp is regarded as “identical?”) and its relevance. For example, even though most of these SVs should not have any biological role, is it possible that they may represent hot spots? Or are they merely following the decreasing curve as theoretically supposed?

Miscellaneous comments:

6. In the reply to another reviewer, the authors stated that further verification of methylation detection from the long reads was necessary, so the related contents were deleted from this paper. However, several papers have already assured the detection power or the limit of the this approach (see https://genomebiology.biomedcentral.com/articles/10.1186/s13059-021-02510-z). Was the sequencing depth of other factor insufficient to be used for the methylation call? The reason of the withdraw should be further specified.

7. In the Introduction section (line 15, page 3), “single-molecule, read-time sequencing (SMAT)” should be “single-molecule real-time sequencing (SMRT)”?

8. Perhaps the most important issue is the data availability. Please make sure all the raw data will be available via the controlled access. I wonder if the data deposition to CNCB is admitted according to the journal policy.

**********

**Have all data underlying the figures and results presented in the manuscript been provided?**

Large-scale datasets should be made available via a public repository as described in the *PLOS Genetics* data availability policy, and numerical data that underlies graphs or summary statistics should be provided in spreadsheet form as supporting information.

Reviewer #2: Yes

Reviewer #3: Yes

**********

PLOS authors have the option to publish the peer review history of their article (what does this mean?). If published, this will include your full peer review and any attached files.

If you choose “no”, your identity will remain anonymous but your review may still be made public.

**Do you want your identity to be public for this peer review?** For information about this choice, including consent withdrawal, please see our Privacy Policy.

Reviewer #2: No

Reviewer #3: **Yes: **Yutaka Suzuki

# Author comment: Author response to Decision Letter 1

**DOI:** 10.1371/journal.pgen.1010514.r004

16 Oct 2022

Attachment Submitted filename: Response.docx Click here for additional data file.

# Peer review: Decision Letter 2

**DOI:** 10.1371/journal.pgen.1010514.r005

**Authors:** David J. Kwiatkowski, Richarda de Voer

8 Nov 2022

Dear Dr Wang,

We are pleased to inform you that your manuscript entitled "Long-read sequencing identifies novel structural variations in colorectal cancer" has been editorially accepted for publication in PLOS Genetics. Congratulations!

Before your submission can be formally accepted and sent to production you will need to complete our formatting changes, which you will receive in a follow up email. Please be aware that it may take several days for you to receive this email; during this time no action is required by you. Please note: the accept date on your published article will reflect the date of this provisional acceptance, but your manuscript will not be scheduled for publication until the required changes have been made.

Once your paper is formally accepted, an uncorrected proof of your manuscript will be published online ahead of the final version, unless you’ve already opted out via the online submission form. If, for any reason, you do not want an earlier version of your manuscript published online or are unsure if you have already indicated as such, please let the journal staff know immediately at plosgenetics@plos.org.

In the meantime, please log into Editorial Manager at https://www.editorialmanager.com/pgenetics/, click the "Update My Information" link at the top of the page, and update your user information to ensure an efficient production and billing process. Note that PLOS requires an ORCID iD for all corresponding authors. Therefore, please ensure that you have an ORCID iD and that it is validated in Editorial Manager. To do this, go to ‘Update my Information’ (in the upper left-hand corner of the main menu), and click on the Fetch/Validate link next to the ORCID field.  This will take you to the ORCID site and allow you to create a new iD or authenticate a pre-existing iD in Editorial Manager.

If you have a press-related query, or would like to know about making your underlying data available (as you will be aware, this is required for publication), please see the end of this email. If your institution or institutions have a press office, please notify them about your upcoming article at this point, to enable them to help maximise its impact. Inform journal staff as soon as possible if you are preparing a press release for your article and need a publication date.

Thank you again for supporting open-access publishing; we are looking forward to publishing your work in PLOS Genetics!

Yours sincerely,

Richarda de Voer

Guest Editor

PLOS Genetics

David Kwiatkowski

Section Editor

PLOS Genetics

www.plosgenetics.org

Twitter: @PLOSGenetics

----------------------------------------------------

Comments from the reviewers (if applicable):

Reviewer's Responses to Questions

**Comments to the Authors:**

**Please note here if the review is uploaded as an attachment.**

Reviewer #2: The authors have addressed all my comments adequately.

**********

**Have all data underlying the figures and results presented in the manuscript been provided?**

Large-scale datasets should be made available via a public repository as described in the *PLOS Genetics* data availability policy, and numerical data that underlies graphs or summary statistics should be provided in spreadsheet form as supporting information.

Reviewer #2: Yes

**********

PLOS authors have the option to publish the peer review history of their article (what does this mean?). If published, this will include your full peer review and any attached files.

If you choose “no”, your identity will remain anonymous but your review may still be made public.

**Do you want your identity to be public for this peer review?** For information about this choice, including consent withdrawal, please see our Privacy Policy.

Reviewer #2: No

----------------------------------------------------

**Data Deposition**

If you have submitted a Research Article or Front Matter that has associated data that are not suitable for deposition in a subject-specific public repository (such as GenBank or ArrayExpress), one way to make that data available is to deposit it in the Dryad Digital Repository. As you may recall, we ask all authors to agree to make data available; this is one way to achieve that. A full list of recommended repositories can be found on our website.

The following link will take you to the Dryad record for your article, so you won't have to re‐enter its bibliographic information, and can upload your files directly: 

http://datadryad.org/submit?journalID=pgenetics&manu=PGENETICS-D-21-01626R2

More information about depositing data in Dryad is available at http://www.datadryad.org/depositing. If you experience any difficulties in submitting your data, please contact help@datadryad.org for support.

Additionally, please be aware that our data availability policy requires that all numerical data underlying display items are included with the submission, and you will need to provide this before we can formally accept your manuscript, if not already present.

----------------------------------------------------

**Press Queries**

If you or your institution will be preparing press materials for this manuscript, or if you need to know your paper's publication date for media purposes, please inform the journal staff as soon as possible so that your submission can be scheduled accordingly. Your manuscript will remain under a strict press embargo until the publication date and time. This means an early version of your manuscript will not be published ahead of your final version. PLOS Genetics may also choose to issue a press release for your article. If there's anything the journal should know or you'd like more information, please get in touch via plosgenetics@plos.org.

# Editor report: Acceptance letter

**DOI:** 10.1371/journal.pgen.1010514.r006

**Authors:** David J. Kwiatkowski, Richarda de Voer

17 Feb 2023

PGENETICS-D-21-01626R2 

Long-read sequencing identifies novel structural variations in colorectal cancer 

Dear Dr Wang, 

We are pleased to inform you that your manuscript entitled "Long-read sequencing identifies novel structural variations in colorectal cancer" has been formally accepted for publication in PLOS Genetics! Your manuscript is now with our production department and you will be notified of the publication date in due course.

The corresponding author will soon be receiving a typeset proof for review, to ensure errors have not been introduced during production. Please review the PDF proof of your manuscript carefully, as this is the last chance to correct any errors. Please note that major changes, or those which affect the scientific understanding of the work, will likely cause delays to the publication date of your manuscript. 

Soon after your final files are uploaded, unless you have opted out or your manuscript is a front-matter piece, the early version of your manuscript will be published online. The date of the early version will be your article's publication date. The final article will be published to the same URL, and all versions of the paper will be accessible to readers.

Thank you again for supporting PLOS Genetics and open-access publishing. We are looking forward to publishing your work! 

With kind regards,

Zsofi Zombor

PLOS Genetics

On behalf of:

The PLOS Genetics Team

Carlyle House, Carlyle Road, Cambridge CB4 3DN | United Kingdom

plosgenetics@plos.org | +44 (0) 1223-442823

plosgenetics.org | Twitter: @PLOSGenetics
